# Practitioner Review: Pathways to care for ADHD – a systematic review of barriers and facilitators

**DOI:** 10.1111/jcpp.12398

**Published:** 2015-02-23

**Authors:** Nicola Wright, Maria Moldavsky, Justine Schneider, Ipsita Chakrabarti, Janine Coates, David Daley, Puja Kochhar, Jon Mills, Walid Sorour, Kapil Sayal

**Affiliations:** ^1^ School of Health SciencesUniversity of NottinghamNottinghamUK; ^2^ Specialist Services DirectorateNottinghamshire Healthcare NHS TrustNottinghamUK; ^3^ School of Sociology and Social PolicyUniversity of NottinghamNottinghamUK; ^4^ Division of PsychologyNottingham Trent UniversityNottinghamUK; ^5^ Division of Psychiatry and Applied PsychologySchool of MedicineUniversity of NottinghamNottinghamUK; ^6^ Child and Adolescent PsychiatryLincolnshire Partnership NHS Foundation TrustLincolnshireUK

**Keywords:** Attention‐deficit disorder with hyperactivity, child, continuity of patient care, health service needs and demands, developmental disability

## Abstract

**Background:**

Attention‐Deficit/Hyperactivity Disorder (ADHD) is a common neurodevelopmental disorder starting in childhood that may persist into adulthood. It can be managed through carefully monitored medication and nonpharmacological interventions. Access to care for children at risk of ADHD varies both within and between countries. A systematic literature review was conducted to investigate the research evidence related to factors which influence children accessing services for ADHD.

**Method:**

Studies investigating access to care for children at risk of ADHD were identified through electronic searches of the international peer‐reviewed and grey literature. Databases were searched from inception till 30th April 2012. This identified 23,156 articles which were subjected to three levels of screening (title, abstract and full text) by a minimum of two independent reviewers. Due to the heterogeneity in the study designs, a narrative approach was used to present the findings.

**Results:**

Twenty‐seven papers met the inclusion criteria; these were grouped into four main themes, with some papers being included in more than one. These were wider determinants (10 papers); identification of need (9 papers); entry and continuity of care (13 papers) and interventions to improve access (4 papers). Barriers and facilitators to access were found to operate at the individual, organisational and societal level. Limited evidence of effective interventions to improve access was identified.

**Conclusion:**

This review explored the multilayered obstacles in the pathway to care for children at risk of ADHD and the lack of evidence‐based interventions designed to address these issues, thereby indicating areas for service development and further evaluative research.

## Introduction

Attention‐Deficit Hyperactivity Disorder (ADHD) is a common neurodevelopmental disorder beginning in childhood and often persisting into adulthood (American Psychiatric Association, 2013). The American Psychiatric Association in the Diagnostic and Statistical Manual of Mental Disorders [DSM V] (2013) states that ADHD occurs in most cultures, affecting approximately 5% of children and 2.5% of adults. In the United Kingdom, within the school age population, there is an estimated overall prevalence rate of 3–9% (NICE, 2008). Childhood ADHD can be managed with carefully monitored medication and nonpharmacological interventions (Brown et al., 2005; Sonuga‐Barke et al., 2013). However, there is evidence that many children with ADHD remain undiagnosed and do not access these interventions (Bussing, Zima, Perwien, Belin, & Widawski, 1998). Left unmanaged, ADHD can result in impairments in multiple domains, including academic performance and parental productivity (e.g. being able to undertake paid employment; Doshi et al., 2012; Shaw et al., 2012). It can also increase the risk of conduct and personality disorders, substance misuse and impaired social adjustment in adulthood (Biederman et al., 2006). Although the lifetime consequences and costs of ADHD are only beginning to be identified (D'Amico et al., 2014; NICE, 2008); the reported impacts of ADHD on children's development, families and society present a strong impetus for early identification, access to services and evidence‐based interventions.

The UK National Institute for Health and Care Excellence (NICE) and the American Academy of Pediatrics both advocate a comprehensive approach to treating ADHD, including multidisciplinary assessment, support for parents and medication for children in moderate or severe cases (American Academy of Pediatrics, 2011; National Institute for Health & Care Excellence, 2008). In an ideal situation, every child who has ADHD would be identified and referred to a specialist and be offered appropriate evidence‐based diagnosis and interventions, including behavioural interventions. In practice, epidemiological studies show that ADHD is underdiagnosed, pathways through services are not clear and therefore access to care is not universal. Bussing, Zima, Perwien, et al. (1998), Bussing, Zima, Gary, and Wilson‐Garvan (2003), Sayal, Goodman, and Ford (2006) and Sayal, Ford, and Goodman (2010) identify that there are huge variations both between and within countries and as a consequence needs remain unmet. Despite this, it should be noted that there are also concerns regarding mis‐diagnosis in some geographical regions. Children may receive a clinical diagnosis even if they do not meet diagnostic criteria for ADHD. For example, a study conducted in North Carolina found that 10% of children had been given an ADHD diagnosis (Rowland et al., 2002).

‘Access to care’ has been identified as a diffuse, diverse and complex phenomenon, incorporating elements of sociology, psychology, management, economics and epidemiology among others (Dixon‐Woods et al., 2006). It has not been consistently defined and there are substantial adjunct literatures, for example quality of healthcare, priority setting and patient satisfaction (Dixon‐Woods et al., 2006). The definition of access to care adopted for this review draws on Gulliford et al. (2001). This model of ‘access to care’ has been used in other literature reviews such as Alborz, McNally, and Glendinning's (2005) review of ‘access to care’ for people with learning disabilities.

Gulliford et al. (2001) conceptualisation identifies that the term ‘access to healthcare’ can be used in two ways: having access and gaining access. Having access refers to the physical existence and availability of a service, whereas gaining access relates to being able to successfully use a service appropriate to need (Gulliford et al., 2001). Gaining access to care for ADHD may be influenced by a number of factors including individual preconceptions about ADHD, availability and affordability of services (and in some countries, insurance status), long waiting lists for services, symptom severity, comorbidity and the knowledge and attitudes of professionals (health and education), parents and young people themselves (Gulliford et al., 2001). Some of these factors such as individual perceptions and the attitudes of professionals may be influenced by the political debate surrounding the conceptualisation of ADHD as a valid entity. Despite the considerable work (consensus activities with experts and a systematic review) to ascertain the validity of the diagnosis of ADHD prior to the development of National Institute for Health and Care Excellence (2008) guidelines, there remains scepticism and misinformation within professional groups (e.g. teachers) which may impact on children gaining access to services (Moldavsky, Groenewald, Owen, & Sayal, 2013).

In this paper, we systematically review qualitative and quantitative evidence on the barriers and facilitators that influence the identification of children at risk of ADHD and their access to services. Given the multidimensional nature of the phenomenon of interest (access to care and ADHD), an approach to reviewing which can accommodate the different spheres of influence operating at multiple levels is required. We chose to adopt a broadly social‐ecological perspective (Susser & Susser, 1996) which seeks to illuminate the social, cultural and individual relations which affect human behaviour to guide our interpretation of the findings in relation to access to care.

This perspective also links to the implementation science agenda, which in broad terms seeks to promote the uptake of research evidence into clinical practice (Eccles et al., 2009). Like the social‐ecological perspective we have used, Woolfe (2008) states that the use of evidence in clinical practice is influenced by a number of different factors operating at different levels, for example individual and organisational. Through the literature review process and the identification of barriers and facilitators to accessing care, we have also aimed to demonstrate where there is evidence which individual clinicians and wider healthcare organisations could implement and also those areas where it is lacking and further work is required.

## Methods

The following databases were searched from inception to the end of April 2012: MEDLINE (PubMed); EMBASE (www.embase.com); PsycINFO; ISI Web of Science; The Cochrane Library (CDSR, CENTRAL, DARE, EE, HTA); OpenSIGLE, System for Information on Grey Literature in Europe (http://opensigle.inist.fr); International Political Science Abstracts (http://iab.sagepub.com/); NHS EED, National Health Services Economic Evaluation Database, CRD (http://www.crd.york.ac.uk/crdweb/ and TRIP database (http://www.tripdatabase.com/). Search terms were developed using keywords for ‘health service barriers’ and ‘ADD/ADHD’ as the relevant terms. The list of search terms is provided in the online supplementary Appendix S1. The initial search was not restricted by language or publication status. In addition, to find information on studies in progress and unpublished or grey literature, the following databases were also searched: World Bank Documents & Reports (http://www-wds.worldbank.org/); OECD (Organization for Economic Co‐operation and Development) Publications & Documents (www.oecd.org); APA PsycNET (http://psycnet.apa.org/); UK Clinical Research Network Database (http://public.ukcrn.org.uk/search/); National Research Register Projects Database (https://portal.nihr.ac.uk/); Health Development Agency (http://www.hda-online.org.uk/); National Primary Care Research and Development Centre (www.npcrdc.man.ac.uk); Children's Society (http://www.the-childrens-society.org.uk/) and relevant American organisations such as the Centre for Disease Control and Prevention. The reference lists of included studies were also scanned to check for further relevant publications.

**Figure 1:**
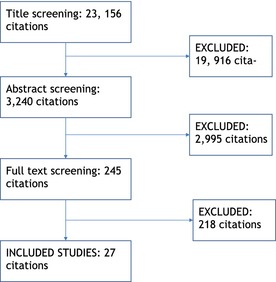
Adapted PRISMA diagram (Moher et al., 2009) showing flow of papers through the review

The searching process generated a total of 23,156 citations for screening. Screening involved three stages: including or excluding studies based on the title only, including or excluding based on the abstract and finally including or excluding based on reading the full text. Each stage was completed by a minimum of two independent reviewers. If necessary, a third person adjudicated in the event of a disagreement. This process and the number of papers included and excluded at each stage are summarised in an adapted version of the PRISMA flow chart (Moher, Liberati, Tetzlaff, & Altman, 2009) shown in Figure 1.

As research evidence on diffuse topics such as ‘access to care’ is likely to reflect a plurality of methods and approaches, Alborz and McNally (2004) and Dixon‐Woods et al. (2006) argue that it is inappropriate to adopt inclusion criteria based only on study design or a hierarchy of evidence. No judgments were made about the relative merits of quantitative versus qualitative research and papers were prioritised on the basis of relevance to the phenomenon of interest rather than particular study types or papers that met particular methodological criteria. Therefore, an evaluation of the methodological quality of the identified studies was not conducted per se. However, the methodology was evaluated in relation to the ability of each study to answer the review questions. Like Alborz and McNally (2004), the inclusion/exclusion process involved balancing the ‘signal’ of the publication (i.e. its potential to illuminate access to care for children with ADHD) against its ‘noise’ or relevant methodology to address the review questions. This means that the methodological rigour of studies was not formally assessed as would be usual within a traditional meta‐analysis or systematic review (e.g. by using the Jadad Scale or a risk of bias tool). Such scales are applicable to the appraisal of the relative quality of RCTs, however this review identified only one RCT relevant to the topic of barriers and facilitators, hence a means of appraisal that permits the synthesis of findings from different methodological approaches was adopted.

Taking this into account, Table 1 summarises methodological inclusion and exclusion criteria applied to the identified studies which address the review questions. It should be noted that papers which focused only on evaluations of clinician knowledge of ADHD, or interventions aimed at its improvement, were not included in this review. Although it may be assumed that knowledge of ADHD influences access to care for children, unless this link was explicitly made in the study findings, the paper was excluded. Papers were also excluded if the sample contained only children with a preexisting clinical diagnosis of ADHD as this implied that they had already accessed services. Studies where a research diagnosis of ADHD (e.g. through screening for eligibility) was applied to a child were included in the review. Papers which had a sample of children with mixed diagnoses were included in the review if a minimum threshold of 50% of the sample had ADHD. This figure was decided upon to ensure that there was sufficient data presented within the paper which was relevant to this review's research question.

**Table 1:** Literature review inclusion and exclusion criteria

	Inclusion criteria	Exclusion criteria
Study design	Empirical studies reporting original data including those employing quantitative (e.g. randomised controlled trials, quasi‐experimental, cross‐sectional surveys, cohort studies and secondary analysis of databases), qualitative (e.g. observation, interviews and focus groups) or mixed methods (e.g. those studies utilising both quantitative and qualitative methods). Extractable data related to ADHD and its symptoms.	Studies which do not include any data. For example narrative reviews, book reviews, commentaries, opinion leader reviews Case studies. Letters, editorials and practice guidelines. No extractable data related to ADHD.
Population of interest	Children or young people (age ≤ 18) at risk of ADHD. Healthcare professionals, teachers, parents or others caring for children at risk of ADHD. Children given a research diagnosis of ADHD. Where studies reported a mixed sample (i.e. children with ADHD and other mental health problems), at least 50% of the sample to have ADHD symptoms.	Adults (over 18 years) with ADHD. Those caring for adults with ADHD, for example healthcare professionals and informal/family carers. Studies evaluating ADHD as a comorbidity of other mental disorders, such as substance abuse or personality disorders. Studies with a sample of less that 50% with ADHD. Children with a clinical diagnosis of ADHD.
Focus of study	Studies which address access to care and reflect the aims of the review.	Studies which do not address access to care or reflect the aims of the review. Service evaluation or satisfaction studies. Studies which focus on the improvement of knowledge in relation to ADHD and its symptoms without making the link to access to care.
Language	Studies reported in the English language.	Studies not reported in the English language.
Period of interest	Studies reported after 1980.	Studies reported before 1980.

Following the three levels of screening, 27 papers were identified for inclusion in the review. Of the 27 included papers, 16 originated in the United States, seven were UK‐based, two were from Australia and there was one each from Greece and Taiwan. Further details of the included studies can be found in Table 2. The reasons for exclusion at the full text stage can be found in the online supplementary Appendix S2.

**Table 2:** Studies Included

Reference	Research methods	Population of interest	Country	Setting	Sample size	Gender	Ethnicity	Child age range	Main conclusions	Review theme
Bussing, Zima, Perwien, et al. (1998)	Cross‐sectional study incorporating two stages: screening using standardised questionnaire and for those eligible structured interviews using validated measures.	Parents and teachers	United States	Schools	Parents of 499 children	26% female	47% ethnic minority, mostly AA	2nd to 4th grade (7–10 years)	Mental health services for children with ADHD should be integrated with general healthcare to address unmet need	Wider Determinants
Bussing, Zima, and Belin (1998)	Cross‐sectional study incorporating two stages: screening using standardised questionnaire and for those eligible structured interviews using validated measures.	Children in special education programmes	United States	Schools	143 children	19% female	45% ethnic minority	2nd to 4th grade (7–10 years)	Primary care clinicians need to be more vigilant in detecting the needs of children in special education programmes who are female, live in rural areas or belong to ethnic minorities	Wider Determinants Entry and Continuity
Bussing, Zima, Gary, and Wilson‐Garvan (2003)	Longitudinal cohort study following up participants in multiple waves using standardised questionnaires following a screening interview.	Parents and teachers	United States	Schools	389 children at risk of ADHD reported (fewer children included in analysis)	48% female	48% C, 52% AA	Kindergarten to 5th grade (3–11 years)	Pressing need to examine the influence of child gender and ethnicity on parental help‐seeking behaviour	Wider Determinants Entry and Continuity Identification of Need
Bussing, Zima, Gary, Mason, et al. 2003	Longitudinal cohort study following up participants in multiple waves using standardised questionnaires following a screening interview.	Parents and children at risk of ADHD	United States	Community	266 children	51% female	67% White	Kindergarten to 5th grade (3–11 years)	Clinicians should assess caregiver strain and social support and integrate them into treatment plans	Wider Determinants
Bussing et al. (2005)	Multilevel design using qualitative interviews and analysis based on grounded theory and standardised questionnaires for deductive analysis to test an application of the network episode model.	Parents of children at risk of ADHD or with ADHD	United States	Community	259 children	52% female	33% AA; the rest C	Kindergarten to 5th grade (3–11 years)	Greater consideration should be given to the influence of gender and ethnicity on the identification and treatment of ADHD	Wider Determinants
Bussing et al. (2011)	Longitudinal cohort study following up participants in multiple waves using standardised questionnaires following a screening interview.	Parents and adolescents	United States	School district in North Florida	168 adolescents	53% female	35% AA	Kindergarten to 5th grade (3–11 years)	Interventions for adolescents with ADHD should include psycho‐education about medication, and should target stigma reduction	Entry and Continuity Identification of Need
Bussing et al. (2012)	Longitudinal cohort study following up participants in multiple waves using standardised questionnaires following a screening interview. Also used grounded theory to analyse open ended survey responses.	Adolescents, parents, healthcare professionals and teachers	United States	School district in North Florida and clinics	569 (148 adolescents)	59% female adolescents	73% C	Kindergarten to 5th grade (3–11 years)	There is a need to develop better strategies to increase adolescents' willingness to engage in treatment for ADHD	Entry and Continuity
Chen et al. (2011)	Cohort study using data from the 1997–2002 National Health Insurance Research Database.	Children and adolescents	Taiwan	Database	10,153 children	23% female	Not reported	Under 17 years	Younger children with ADHD may benefit from mental health services that address socioeconomic and organisational influences on access to care	Entry and Continuity
Cuffe et al. (2009)	Cross‐sectional study using data from the National Health Interview Survey Child and Person level components for 2001.	Parents of children at risk of ADHD	United States	Community	278 children	30% female	Not reported	4–17 years	More intervention provision is required for low‐income families and those living in rural areas	Wider Determinants Entry and Continuity
Gardner et al. (2004)	Cross‐sectional survey	Parents and primary care clinicians	United States	Primary care	659 children at risk of ADHD	22% female	14% ethnic minority	4–15 years	Children at risk of ADHD need more follow‐up visits	Wider Determinants Entry and Continuity
Gidwani et al. (2006)	Vignette based cross‐sectional survey	Parents (mothers)	United States	Primary care	135 mothers	100% of respondents were female	Anglo (in United States for more than 2 generations; *n* = 43); Puerto Rican (*n* = 43); Central or South American (*n* = 49)	6–12 years	Clinicians should not allow parental language competence to be a barrier to discussing children's behaviour	Wider Determinants
Groenewald et al. (2009)	Vignette based postal questionnaire	Primary school teachers	United Kingdom	Community	212 teachers	89% of respondents were female	Not reported	Primary School (4–11 years)	Training about ADHD for teachers may address gaps in their knowledge about ADHD and its treatment	Identification of Need
Hillemeier et al. (2007)	Secondary analysis of data collected as part of a longitudinal study of children at risk off emotional and/or behavioural problems.	Parents	United States	Community	1070 children	36% of the index children were female	51% AA, 49% C	Not stated	Clinicians need to be aware of the influence of ethnicity on parents' recognition of ADHD	Wider Determinants
Larson et al. (2011)	Cross‐sectional analyses conducted on data from the 2007 National Survey of Children's Health	Parents of children at risk of ADHD	United States	Community	5028 children	Not reported	Not reported	6–17 years	Greater consideration should be given to comorbidities in ADHD	Entry and Continuity
Maniadaki et al. (2006)	Questionnaire study using vignettes.	Parents of children aged 4 to 6 years	Greece	Community	590 parents	50% mothers	Not reported	4–6 years	Clinicians need to give greater consideration to the early identification of ADHD	Identification of Need
Morley (2010)	Web‐based factorial survey using vignettes	Family physicians and paediatricians	United States	Primary care	187 clinicians	49% female	88% White	Not stated	Clinicians need to be aware of the potential influence of ethnicity and insurance status on their decision‐making	Identification of Need
Ohan and Visser (2009)	Analogue study using vignettes	Parents of children at risk of ADHD and elementary school teachers.	Australia	Community	96 parents, 140 teachers	Not reported for parents; 85% female for teachers	Parents 88% White; teachers 91% White	Elementary school (6–12 years)	Services need to educate teachers and parents about the effectiveness of treatments for ADHD for both boys and girls	Identification of Need
Sawyer et al. (2004)	Analysis of cross‐sectional data collected for the Child and Adolescent Component of the National Survey of Mental Health and Well‐being.	Parents of children and adolescents at risk of ADHD	Australia	Community	398 parents	Not reported	Not reported	6–17 years	A minority of participants at risk of ADHD received help for their problems. Practical issues, including the cost of services and waiting lists were the most common barriers cited by parents	Entry and Continuity
Sayal et al. (2002)	Cross‐sectional community study	Children at risk of ADHD	United Kingdom	Community	127 children	24% female	Not reported	5–11 years	Parents are the main gatekeepers for access to specialist services for ADHD	Entry and Continuity Identification of Need
Sayal et al. (2003)	Cross‐sectional community survey	Parents of children at risk of ADHD	United Kingdom	Community	93 parents	Not reported	Not reported	5–11 years	The impact of ADHD symptoms on parents' work and family finances is substantial and influences access to care	Entry and Continuity Identification of Need
Sayal, Goodman, and Ford (2006)	Analysis of data collected by the Office for National Statistics in a national single stage cross‐sectional survey	Children with ADHD and their parents and teachers	United Kingdom	Community	232 children	19% female	Not reported	5–15 years	There is a need for health service input to support educational professionals in their contact with concerned parents	Entry and Continuity
Sayal, Hornsey, et al., 2006	Cohort study using a before and after design.	Teachers and children at risk of ADHD	United Kingdom	Schools	96 teachers and 2672 children	50% female	Not reported	Primary School (4–11 years)	A brief educational intervention for teachers could improve the identification of undiagnosed children with ADHD	Interventions
Sayal, Ford, and Goodman (2010)	Analysis of data collected in the 2nd national cross‐sectional survey (British Child and Adolescent Mental Health Survey)	D Children with ADHD and their parents and teachers	United Kingdom	Community	176 children	Not reported	Not reported	5–16 years	There is a need for greater support for schools, to enhance their role in helping parents of children at risk of ADHD	Entry and Continuity
Sayal, Owen, et al., 2010	Population based follow‐up study of a randomised school‐based intervention	Children at risk of ADHD	United Kingdom	Schools	487 children	Not reported	Not reported	4–5 years	There may be adverse effects associated with labelling children at a young age on teachers' perceptions of children's behaviour	Wider Determinants Interventions
Wasserman et al. (1999)	Prospective cohort study.	Primary care physicians	United States	Primary care	401 primary care clinicians reporting on 1947 children having attention and hyperactivity problems	Not reported for the population of interest	Not reported for the population of interest	4‐15 years	Clinicians do not appear to be predisposed to label children as having ADHD‐type problems on the basis of their ethnicity and SES	Identification of Need
Williams et al. (1993)	Cohort study	School nurses and children at risk of ADHD	United States	Schools	110 children	Not reported	Not reported	Not stated	Authors conclude that the intervention helped improve the educational life of the children	Interventions
Wolraich et al. (2005)	Randomised control trial	Primary care physicians, teachers, parents and children at risk of ADHD	United States	Primary care and schools	243 children	31% female	56% AA, 40% C	Kindergarten to 4th grade (5–10 years)	Interventions aimed at improving communication should be continuous	Interventions

AA, African‐American; C, Caucasian.

It has already been noted that ‘access to care’ is a diffuse concept and is open to multiple interpretations. Drawing on the social‐ecological view of research implementation and the model developed by Gulliford et al. (2001) to illustrate the interaction between different factors affecting access to care, the included papers were grouped into four main themes. As some factors span more than one theme (e.g. adult perceptions), there is sometimes a degree of overlap between themes. We have aimed to minimise repetition to aid the flow of the review for clinical practitioners. The four themes were: Wider Determinants (10 papers), Identification of Need (8 papers), Entry and Continuity of Care (13 papers) and Interventions to Improve Access (4 papers). Some studies were assigned to more than one theme as they explored multiple facets of ‘access to care’. The Wider Determinants, Identification of Need and Entry and Continuity themes are described within the Gulliford et al. (2001) model. Papers within the Wider Determinants theme referred to the effects of sociodemographic characteristics such as gender, ethnicity and socioeconomic status on access to care. Although not readily amenable to change by healthcare services, these factors may influence how behaviours are interpreted by parents, teachers or healthcare professionals and therefore impact on a child's access to services for ADHD. Recognition of a need for a service is often the first step in accessing healthcare and for children at risk of ADHD the initiative is likely to come from key adults such as parents and teachers. The papers categorised within the *Identification of Need* theme explored the roles of individual‐level factors (including adult perceptions and social resources) in influencing the help‐seeking process. Studies within the *Entry and Continuity* theme focused on clinical predictors of service utilisation and the role of professionals as gatekeepers to appropriate specialist services. In addition to using the Gulliford et al. (2001) categories, it was recognised that successful access to services for young people with ADHD may rely on novel approaches to care provision. The review also therefore included papers which evaluated interventions aimed at improving access to care (*Interventions to Improve Access*).

The wide heterogeneity in study design meant that a meta‐analysis of the results was not possible and therefore the findings from the review are presented as a narrative under each theme heading. Although this approach has been taken, where they have been reported and add to the interpretation and synthesis of the papers, odds ratios and confidence intervals are also presented.

## Findings

### Wider determinants

Wider determinants are those barriers and facilitators to accessing care which relate to societal or macrolevel influences. Ten papers were identified within this theme with nine coming from the United States (Bussing, Koro‐Ljungberg, Gary, Mason, & Garvan, 2005; Bussing, Zima, & Belin, 1998; Bussing, Zima, Perwien, et al., 1998; Bussing, Zima, Gary, & Wilson‐Garvan, 2003; Bussing, Zima, Gary, Mason, et al. 2003; Cuffe, Moore, & McKeown, 2009; Gardner, Kelleher, Pajer, & Campo, 2004; Gidwani, Opitz, & Perrin, 2006; Hillemeier, Foster, Heinrichs, & Heier, 2007). These papers highlighted the roles of child gender (Bussing, Zima, Perwien, et al., 1998; Bussing, Zima, Gary, & Wilson‐Garvan, 2003; Bussing et al. 2005; Sayal, Owen, et al., 2010), age (Gardner et al., 2004), ethnicity (Bussing, Zima, Perwien, et al., 1998; Bussing, Zima, Gary, & Wilson‐Garvan, 2003; Bussing et al. 2005; Gidwani et al., 2006; Hillemeier et al., 2007), social networks (Bussing, Zima, Gary, Mason, et al. 2003), low socioeconomic status (Bussing, Zima, Perwien, et al., 1998; Bussing, Zima, Gary, et al. 2003; Cuffe et al., 2009; Gardner et al., 2004) and urban residence (Bussing, Zima, & Belin, 1998; Cuffe et al., 2009) as factors influencing access to care for children at risk of ADHD.

Bussing, Zima, Perwien, et al. (1998), Bussing, Zima, and Belin (1998), Bussing, Zima, Gary, and Wilson‐Garvan (2003), Bussing, Zima, Gary, Mason, et al. (2003) and Bussing et al. (2005) in the United States (Florida) investigated the relationship between gender and ethnicity and access to care for children at risk of ADHD. The first set of studies (Bussing, Zima, Perwien, et al., 1998; Bussing, Zima, Belin, et al., 1998) screened a sample of 499 children (mean age 9 years) receiving special education (defined as either the presence of a specific learning disability or emotional handicap) in a school district. Diagnostic assessments for ADHD were carried out for 207 ‘high‐risk’ children who scored above a cut‐off point on parent questionnaires or had received treatment for ADHD. These were also completed on a random sample of 200 controls (Bussing, Zima, Perwien, et al., 1998). ‘Unmet need’ for ADHD care was defined as meeting diagnostic criteria without having received ADHD treatment in the last year. This applied to half the group and was associated with female gender (OR 3.0, *p* < .001) and ethnic minority status (OR 2.0, *p* < .001; Bussing, Zima, Perwien, et al., 1998).

Bussing, Zima, Gary, and Wilson‐Garvan (2003), Bussing, Zima, Gary, Mason, et al. (2003) also found that there were gender and ethnic disparities in terms of recognition, help‐seeking and service use for children with ADHD. They screened district school records to identify children with ADHD risk predictors: 3,158 students were selected in a stratified random design which oversampled girls by a margin of two to one. Telephone interviews were conducted with 1,615 parents of identified children and from this group two samples were selected: 389 children for analysis of help‐seeking and 91 for analysis of access to care barriers (Bussing, Zima, Gary, & Wilson‐Garvan 2003). Gender and ethnicity were not found to independently affect the rate of recognition of children at risk of ADHD; however, both variables had a consistent effect on the subsequent help‐seeking steps of evaluation, obtaining a diagnosis and receiving treatment. In each of these three steps, boys were found to be five times more likely to access services and receive help than girls (OR 5.8, CI 3.4–10.0; OR 5.4, CI 3.0–9.6; OR 5.5, CI 2.8–10.7 respectively). White children were twice as likely to access services as African‐American children (OR 2.9; CI 1.6–5.2, OR 2.8; CI 1.5–5.1, OR 2.2; CI 1.1–1.3, respectively for the 3 steps) Bussing, Zima, Gary, and Wilson‐Garvan (2003). As well as identifying variations in and predictors of accessing services, Bussing, Zima, Gary, and Wilson‐Garvan (2003) also sought to identify parental perceptions of barriers to care. The total number of barriers identified was not found to vary by gender or ethnicity. However, the parents of girls reported more stigma‐related barriers in comparison to boys, and African‐American parents expressed more negative expectations of treatment than their White peers (Bussing, Zima, Gary, & Wilson‐Garvan 2003).

Possible explanations for these differences were explored by Bussing, Zima, Gary, Mason, et al. (2003), Bussing et al. (2005). The influence of social networks in terms of their structure, size and type of support offered was investigated by Bussing, Zima, Gary, Mason, et al. (2003). Although child gender did not impact on network characteristics; ethnicity and socioeconomic status did lead to significant variation. The networks of White and higher socioeconomic parents were found to be significantly larger (*p* < .0001) and contain a higher proportion of healthcare professionals (*p* < .001) (Bussing, Zima, Gary, Mason, et al. 2003). This suggests that these groups are more able to access advice from professionals, both formally and informally, than others. Despite a lack of healthcare professionals within their networks, African‐American parents and those from lower socioeconomic groups did not appear to lack access to informal support, as they reported more frequent contact with relatives and friends, with greater perceived affective (*p* < .001), affirmative (*p* < .001) and instrumental (*p* < .0001) support.

In a subsequent paper, Bussing et al. (2005) explored explanations for gender and ethnic differences in help‐seeking behaviours; using qualitative methods and further analysis of the Bussing, Zima, Gary, and Wilson‐Garvan (2003) data set. They found that parental conceptualisations of a child's behaviour were influenced by gender and ethnicity. For example, African‐American girls were conceptualised as a ‘misbehaving child’ (characterised by behavioural problems), whereas African‐American boys were described as ‘endangered’ (requiring close supervision to prevent harm). These differing conceptualisations were found to influence the help‐seeking behaviour of parents. For example, parents reported making fewer steps (and therefore attempts) to seek help for girls in comparison to boys and also for African‐American as compared to White children (Bussing et al., 2005). A UK‐based intervention study (discussed in more detail in the Interventions to Improve Access theme) also confirmed gender differences in relation to access to care. In this 5‐year follow‐up study, Sayal, Owen, et al. (2010) found that male children at risk of ADHD were more likely to access specialist health services.

Two studies in the United States focused specifically on the influence of ethnicity on parental perceptions of symptoms of ADHD. In a vignette study, Gidwani et al. (2006) investigated the differences between Anglo (defined in this study as being in the United States for more than two generations) and Latino mothers' perceptions of ADHD behaviours. Differences were found based on whether mothers were Spanish or English speakers. For example, Spanish speakers were less likely to rate vignettes describing a child with behaviour compatible with ADHD as ‘normal’. They also expressed a greater interest in discussing the child's behaviour with physicians (Gidwani et al., 2006). Exploring variation in parental report of ADHD symptoms, Hillemeier et al. (2007) found that African‐American and White parents endorsed different items on the Diagnostic Interview Schedule for Children (DISC). This was despite similar levels of need in the child. Items related to hyperactivity, impulsivity and concentration were endorsed more frequently for African‐American children, while parents of White children were more likely to endorse items related to organisational problems at home. Both the Gidwani et al. (2006) and Hillemeier et al. (2007) studies indicate that cultural and language variables may play a role in access to care for ADHD.

The Bussing, Zima, Perwien, et al. (1998), Bussing, Zima, and Belin (1998), Bussing, Zima, Gary, and Wilson‐Garvan (2003) studies also highlighted the roles of other sociodemographic factors which impact on access to care for ADHD. Low socioeconomic status (based on measures reflecting insurance and subsidised lunch status) was identified as an indicator of unmet need, approximately doubling the odds that a child with ADHD would not receive services (Bussing, Zima, Perwien, et al., 1998). This was supported by findings from Bussing, Zima, Gary, and Wilson‐Garvan (2003) which suggested that the likelihood of receiving ADHD treatment was higher for ‘nonpoor’ children in comparison to their ‘impoverished’ peers (OR 2.8, 95% CI) (Bussing, Zima, Gary, & Wilson‐Garvan, 2003). Higher rates of service use in the general health sector were associated with being an urban resident (Bussing, Zima, & Belin, 1998). These findings were also supported by Cuffe et al. (2009) drawing on data from the SDQ (Strengths and Difficulties Questionnaire) component of the 2001 National Health Interview Survey and by Gardner et al. (2004) who used data from the US primary care‐based Child Behavior Study. Cuffe et al. (2009) also found that younger age was associated with access to care for ADHD – children between the ages of 9 and 13 were more likely to report a visit to a medical care professional than 14‐ to 17‐year olds (OR 2.77, CI 1.32–5.81).

### Identification of need

Adult recognition that a child has difficulties and identification of a need for services are fundamental steps for children to be able to access appropriate care. Seven studies discussed elements related to the identification of need and, of these, three were from the United States (Bussing, Zima, Gary, & Wilson‐Garvan 2003; Morley, 2010; Wasserman et al., 1999), two from the United Kingdom (Groenewald, Emond, & Sayal, 2009; Sayal, Taylor, Beecham, & Byrne, 2002) and one each from Australia (Ohan & Visser, 2009) and Greece (Maniadaki, Sonuga‐Barke, Kakouros, & Karaba, 2006). What adults believe about the nature of ADHD, the potential benefits of accessing services and how they conceptualise behavioural and emotional difficulties can all be influential, whether they are teachers (Groenewald et al., 2009; Ohan & Visser, 2009), parents (Bussing, Zima, Gary, & Wilson‐Garvan 2003); Maniadaki et al., 2006; Ohan & Visser, 2009) or clinicians (Morley, 2010; Sayal et al., 2002; Wasserman et al., 1999).

Using vignette descriptions of a girl with ADHD, Groenewald et al. (2009) explored teacher recognition and, in particular, the effect of the ADHD subtypes. How teachers conceptualised difficulties rather than the actual differences in symptomatology was found to influence teacher recognition and referral to specialist services (Groenewald et al., 2009). Results from a multivariable analysis demonstrated that conceptualisation of problems increased the propensity for referral, whether seen as ‘emotional difficulties’ (by 6%, 95% CI 0–11%; *p *=* *.042) or ADHD (by 14%, 95% CI 8–21%; *p *=* *.0001). By contrast, conceptualisation as ‘attentional difficulties' reduced the teacher's likelihood of making a referral by 13% (95% CI 5–21%; *p *=* *.002; Groenewald et al., 2009).

In a study of parents of preschool children, some of whom presented with ADHD behaviours, Maniadaki et al. (2006) found that parents whose child displayed ADHD characteristics conceptualised the behaviour of the child in a vignette as less severe than parents with a child with less ADHD symptoms (*p* = .05). Accordingly, parents with a child with ADHD symptoms perceived the behaviour of the child as having less impact on his/her life than the other group of parents (*p* = .001). Maniadaki et al. (2006) inferred that, even though parents of preschool children acknowledge the need to seek specialist help in the case of a child with ADHD‐related difficulties, in practice they usually fail to recognise the presence and clinical meaning of ADHD behaviours in their own children.

Reduced likelihood of referral for girls was also investigated by Ohan and Visser (2009) using a sample of parents and teachers. In contrast to both Groenewald et al. (2009) and Maniadaki et al. (2006), referral was not found to be linked to perceptions and conceptualisations of child behaviour (in this case, disruption) but was instead linked to beliefs about the potential of a child to benefit from interventions such as learning assistance. Using the example of learning assistance, Ohan and Visser (2009) found that both parents (*p* < .01) and teachers (*p* < .01) reported that it would be more beneficial to boys than girls. They suggested that this belief system influenced parents' and teachers' decision‐making, and mediated the relationship between gender and service seeking (Ohan & Visser, 2009). The strongest predictors of General Practitioner (GP) recognition of problems in a study in the United Kingdom were found to be parental recognition and request for referral (OR 20.83, 95% CI 3.05–142.08), in conjunction with behavioural problem comorbidity (OR 1.48 95% CI 1.04–2.12; Sayal et al., 2002). In turn, GP recognition of a mental health disorder invariably led to a likely referral to specialist mental health services, suggesting that GP nonrecognition constitutes a barrier in the pathway to care.

Morley (2010) and Wasserman et al. (1999) also investigated primary care clinicians' recognition, diagnosis and treatment patterns in relation to ADHD. Neither study found evidence that ADHD is used by GPs as a label for children with social and family problems. Although there were some gender differences in recognition and diagnosis of symptoms (e.g. boys were more likely than girls to be regarded as having attention and hyperactivity problems), the predominant finding in both studies (using different methodologies) was that a positive ADHD status was most influential in determining recognition (Morley, 2010; Wasserman et al., 1999).

### Entry and continuity of care

A number of papers explored issues pertaining to take‐up of consultations with clinical services, or service utilisation, although we did not include papers about treatment adherence over the longer term. Findings from studies conducted in Australia (Sawyer et al., 2004), the United Kingdom (Sayal, Taylor, & Beecham, 2003; Sayal, Taylor, Beecham, & Byrne, 2002; Sayal, Goodman, Ford, 2006, 2010), the United States (Bussing, Zima, Mason, Porter, & Garvan, 2011; Bussing, Zima, Perwien, et al. 1998; Bussing, Zima, & Belin 1998; Bussing et al., 2012; Cuffe et al., 2009; Gardner et al., 2004; Larson, Russ, Kahn, & Halfon, 2011) and Taiwan (Chen et al., 2011) point to a number of factors which influence service utilisation. These include: comorbid disorders, adult perceptions (including parental recognition of problems and parent‐reported burden), willingness to engage (by parents, teachers, healthcare professionals and adolescents) and organisational issues. Further details are presented below.

Parental perceptions of hyperactivity as a serious problem requiring help and child emotional and behavioural comorbidities have been shown to predict use of specialist mental health services in UK studies conducted by Sayal, Taylor, Beecham, and Byrne (2002), Sayal, Taylor, and Beecham (2003), Sayal, Goodman, and Ford (2006), Sayal, Ford, and Goodman (2010) and in Australia by Sawyer et al. (2004). Research conducted in the United States by Cuffe et al. (2009), Gardner et al. (2004) and Bussing, Zima, and Belin (1998) supported these findings. In the Cuffe et al. (2009) study, children with high levels of comorbid emotional problems were more likely to be seen by medical professionals than children without (OR 1.98, CI 1.07–3.66). Similarly, in the Gardner et al. (2004) study, the presence of comorbid internalising symptoms was associated with visiting a mental health specialist. Larson et al. (2011) identified that health and education service use increased with each additional comorbid condition in children with symptoms compatible with ADHD. Using the example of mental health visits, Larson et al. (2011) identify that the odds of service use increased by 1.33 for a single comorbidity, 2.73 for two and 4.55 if three were present.

Research by Bussing, Zima, and Belin (1998) found that, in relation to children at high risk of ADHD, receipt of specialist mental health services in the previous year was associated with comorbid behaviour problems, functional impairment and family burden, and it is reasonable to assume that the service use followed the recognition of a problem rather than vice‐versa. In a later study, Bussing et al. (2011) found that parental perceptions of inattention symptoms (OR 1.2 CI 1.05–1.31) and medication receptivity (OR 3.8 CI 1.62–8.71) were significant predictors of mental health service usage in the past year. However, this study also found that the perceptions of older children and adolescents have an equally powerful effect on accessing services. For example, having a ‘medication receptive’ parent increased the odds of using mental health services by 3.8, but perceived stigma on the part of the adolescent reduced these odds by a factor of five (Bussing et al., 2011).

Bussing et al. (2012) expanded on the role of adolescent opinion in a mixed methods enquiry. The perspectives of four sets of stakeholders: teachers (*n* = 122), parents (*n* = 161), healthcare professionals (*n* = 138) and adolescents (*n* = 148) were explored in relation to the reasons individuals gave for wanting to engage with services for ADHD. Parents and healthcare professionals were more willing to engage than adolescents (beta estimates of 0.55 and 0.44 respectively). Other factors that increased willingness to engage with services included feeling knowledgeable about the treatment options (medication and psychosocial) available and a consideration that the treatment (medication or psychosocial) was both acceptable and helpful (Bussing et al., 2012). Willingness was found to be reduced by the anticipation of negative side effects but not significantly related to stigma, embarrassment, ethnicity, gender or socioeconomic status (Bussing et al., 2012).

In contrast to the methodology of the above studies which focused on individual factors and initial access, Chen et al. (2011) suggested that service provider characteristics were the main factor influencing the failure to continue to access services (indicated by the discontinuation of treatment). Although sociodemographic factors played a significant role in the initiation of treatment (being male, lower socioeconomic status and an older age all increased an individual's chances of being treated), they were not found to influence discontinuation. Factors such as where an individual receives treatment (district hospital/clinic vs. paediatric or psychiatric specialists) or a change in hospital or clinic increased the likelihood of discontinuation (Chen et al., 2011). For example, children who received their initial prescription from a district hospital were 1.32 times more likely to discontinue it (95% CI: 1.17–1.49) than those who received their prescription from a specialist paediatrician or psychiatrist.

### Interventions to improve access to care

Only four papers, two UK‐based (Sayal, Hornsey, Warren, MacDiarmid, & Taylor, 2006; Sayal, Owen, et al., 2010) and two from the United States (Williams, Horn, Daley, & Nader, 1993; Wolraich, Bickman, Lambert, Simmons, & Doffing, 2005) reported interventions which aimed to increase access to care for children at risk of ADHD. Three of these studies explored school‐based interventions (Sayal, Hornsey, et al., 2006; Sayal, Owen, et al., 2010; Williams et al., 1993) whereas Wolraich et al. (2005) tested an intervention which aimed to improve communication between professional and family carers.

Sayal, Hornsey, et al. (2006) delivered an ADHD educational session to 96 teachers in six primary schools to investigate changes in recognition of children with probable ADHD. The intervention improved agreement between teacher recognition of possible or probable ADHD and the diagnostic algorithm (based on parent and teacher responses to the SDQ). The sensitivity of teacher recognition was 32% at baseline and 50% after the session; specificity was 97% at baseline and 96% following the session (Sayal, Hornsey, et al., 2006). The authors suggested that a brief educational intervention for teachers could help improve the identification of children with ADHD in the community (Sayal, Hornsey, et al., 2006).

The impact of school‐based screening and educational interventions on longer term outcomes including specialist service use was investigated by Sayal, Owen, et al. (2010). Schools received one of the four interventions: education (books about ADHD for teachers), identification (the names of children with high hyperactivity/inattention scores between the ages of 4 and 5 years), both education and identification, or no intervention. None of the interventions were associated with improved outcomes, and there was no association between intervention type and specialist service use at 5‐year follow‐up. However, children who received the identification‐only intervention were more than twice as likely as those in the control group to have high hyperactivity/inattention scores on the SDQ at follow‐up (adjusted OR 2.11 95% CI 1.12–4.00), indicating a possible association between awareness or labelling of hyperactivity/inattention problems and worse outcomes.

Williams et al. (1993) also evaluated a school‐based intervention. However, unlike Sayal, Hornsey, et al. (2006), Sayal, Owen, et al. (2010) who looked at teacher recognition, Williams et al. (1993) explored the school setting as a means of coordinating the many different services involved in the care for children with ADHD. However, a lack of engagement from parents (35% of parents failed to engage) and insufficient information collected at follow‐up (48 out of a total of 96 children had insufficient information) made robust evaluation of the intervention problematic.

Wolraich et al. (2005) investigated the implementation of an intervention which aimed to improve communication among primary care physicians, teachers and parents who supported children at risk of ADHD. The intervention comprised group workshops, but low up take led to the redesign of the intervention and single one‐to‐one tutorials were offered. While the individual approach was better attended than the workshops, it was not possible to demonstrate improved communication between primary care physicians, teachers and parents (Wolraich et al., 2005). The inference drawn was that a sustained and continuous approach was needed to improve communication between all those involved in caring for children at risk of ADHD. The authors suggest that school‐based mental health services could be a more effective approach, and highlight the potential role of school nurses as facilitators of the communication between teachers and primary care physicians.

## Discussion

### Strengths and limitations of the review process

In this systematic review, the majority of the 27 included papers focused on the characterisation of existing barriers and facilitators within the pathway to care and unmet need within the population, with only four studies investigating interventions aimed at improving access to care for children at risk of ADHD. This review has been informed by both a social‐ecological view of research implementation and the Gulliford et al. (2001) conceptualisation of ‘access to care’. The use of any specific theoretical framework or conceptualisation can be a potential limitation. In this case, using a different theoretical lens may lead to the themes being interpreted in different ways or different themes being identified at the outset. For example, the wider determinants category has emphasised the role of sociodemographics and economic status on access to care as per the Gulliford et al. (2001) model. Using a different theoretical lens, may have meant that the social, regulatory, policy or political landscape that influences the availability and access to services took a more prominent focus.

To attempt to mitigate this, we have presented the review process as transparently as possible so that our decision‐making is clear, and practitioners and policy‐makers are able to make their own judgments about the transferability of the findings to their clinical areas. As outlined in the Methods section, we did not conduct an estimation of methodological rigour as would be expected in more traditional systematic reviews and this may be considered to be a limitation of the review process. Instead, we have focused on the ability of the identified studies to answer the review questions, a precedent set by Alborz et al. (2005). We have noted methodological limitations of the studies where relevant, however given the paucity of included studies this did not inform the weight we attached to their findings or the consideration given to the studies within our results. Therefore, an inclusive approach to the searching and retrieval of studies was undertaken and independent reviewers were used to ensure sufficient rigour within the process.

Although the majority of the included studies have come from the United States, the review has taken an international perspective with studies from Australia, Greece, Taiwan and the United Kingdom. The differential rates of recognition and diagnosis of ADHD between countries as well as variations in healthcare systems may have influenced the barriers and facilitators identified in the review. These societal and system factors can make comparisons across different countries challenging. It may also mean that some of the research findings are specific to the communities in which the original studies were conducted and caution may need to be applied when implementing them in different contexts. Despite the international focus of the literature review, a limitation is that it only reports on studies published in English. It is acknowledged that this approach may have missed key texts.

### Discussion of the review findings

This literature review has utilised a social‐ecological view of implementation and the Gulliford et al. (2001) framework to identify a complicated network of actors involved in accessing care for ADHD. These individuals include parents, teachers, healthcare professionals (specialist and nonspecialist) as well as the children and young people themselves. At this individual level, parental decision‐making in relation to accessing services is influenced by the views of the child's teacher and their ability to recognise behaviours suggestive of ADHD. Beliefs and perceptions regarding the efficacy of treatment and what constitutes acceptable behaviour were also put forward as factors influencing the recognition of ADHD and subsequent help‐seeking behaviours. The child's gender and ethnicity appear to exert an influence on accessing services, as demonstrated by Bussing, Zima, Gary, Mason, et al. (2003), whereby girls and African‐American children were less likely to be recognised as having ADHD. Cultural differences were noted in parental thresholds for deeming behaviour to be problematic (Gidwani et al., 2006), and comorbidity was identified as a strong predictor of access to care.

As well as individual‐level factors, organisational settings as both a place for identification and intervention were considered within the review. Schools, in particular, were investigated in three of the four included intervention studies (Sayal, Hornsey, et al., 2006; Sayal, Owen, et al., 2010; Williams et al., 1993). Improvements in identification were noted, but the impact of identification may have proved detrimental to some children (Sayal, Owen, et al., 2010). The detrimental impact of being identified, and potentially labelled, as having ADHD, raises the issue of stigma and the possibility of a self‐fulfilling prophecy in relation to adult expectations of a child's behaviour. This suggests that recognition and identification may only lead to positive outcomes for the child if they then progress to the receipt and acceptance of evidence‐based interventions. It also raises the question of whether children whose ADHD does not present complex difficulties benefit from being identified. In summary, the impact of ADHD on functioning in educational settings potentially makes school‐based interventions desirable, with the caveat that the threshold for identification should be sufficiently sensitive and specific and the treatment effective enough so that any negative consequences for the child are offset by the advantages gained from care. Single interventions aimed at enhancing the communication among key adults who care for children at risk of ADHD do not appear to be effective in the long term; interventions probably need to be continuous and sustainable (Wolraich et al., 2005).

Although this review has focused on ADHD, individual and organisational barriers and facilitators to care have been identified for a range of paediatric conditions. These include asthma (Lakhanpaul et al., 2014), learning disabilities (Bhaumik et al., 2011) and diabetes (Powell, Chen, Kumar, Streisand, & Holmes, 2013). As found in this review, demographic factors, parental beliefs in the efficacy of treatments and how care is organised are important barriers to care (Lakhanpaul et al., 2014; Liptak et al., 2008; Powell et al., 2013). However, unlike many other childhood conditions, there is a wider political dimension to the existence/nonexistence of ADHD. As identified above, the possibility of creating self‐fulfilling prophecies in relation to young children and their behaviour is concerning for many. Misconceptions regarding ADHD can have a detrimental impact on children and their families. The risk of stigma was raised as an issue for adolescents in the Bussing et al. (2012) study and stigmas negatively impacted on their willingness to access and engage with care. This suggests that there is a need for psycho‐educational interventions for adolescents. Mental health practitioners need to be aware of the importance of adolescents' perceptions of public stigma of ADHD as a barrier to their engagement in the assessment process and also as a factor that decreases their treatment adherence. This finding also identifies the important role of public awareness initiatives and health‐promotion strategies to increase the wider understanding of ADHD within society as starting points for improving access to care. Given the possibility of differing perceptions and explanations for a child's behaviour, building a common understanding of the child's difficulties in shared decision‐making and the pathway through services is crucial.

In summary, this literature review has identified barriers and facilitators to accessing care at multiple levels of influence and how they appear to affect the complex process of diagnosis and treatment for children with ADHD symptoms. Clinicians working with children and adolescents will benefit from an increased awareness of these barriers, to inform any attempts to improve equity in access to care. The studies included in this review suggest that clinicians should evaluate their patients in context, taking into account their age, gender, ethnicity, socioeconomic status, social networks that influence them and their views on possible stigma associated with a diagnosis of ADHD.

The review has also highlighted large gaps in the evidence base, particularly in relation to interventions to improve access. Only a few trials, based in widely differing systems of healthcare, have been conducted and none of these has been replicated, so the little evidence which does exist is not generalisable.

This dearth of research into how best to overcome the numerous barriers to treatment of ADHD is problematic for policy‐makers seeking to improve outcomes for children across the board (Department of Health, 2013) as well as for practitioners aiming to implement evidence‐based practice and clinical guidelines in the field of mental disorders of children and adolescents. However, some of the studies do provide useful ideas about ways forward, for example, the need for a better integration of health and education services (Wolraich et al., 2005).

Timely and effective treatment of ADHD could afford long‐term savings (Lucas et al., 2013) with better knowledge of how to overcome the barriers to access discussed here. More work is therefore needed to evaluate interventions to enhance access to care for children at risk of ADHD. The use of randomised controlled trial methodologies, replication studies and long‐term follow‐up are important means to determine the cost‐effectiveness of such initiatives.

**Key points**

- For children at risk of ADHD, there are barriers and facilitators to the receipt of assessment and eventual treatment; these operate at a societal, organisational and individual level.
- There are inequalities in the access to care for ADHD: boys, younger children, White children, urban residents and children from higher SES are more likely to access services. This bias may be partially mediated by the influence of the child's gender on parent conceptualisation of the problem and variations in help‐seeking behaviour which are associated with ethnicity.
- There is a need to enhance the knowledge about ADHD in teachers, primary care clinicians and parents with special focus on awareness that ADHD might have a predominantly inattentive presentation and that treatment for ADHD is as effective in girls as in boys.
- Adolescents with ADHD may be aware of stigma and public perceptions in relation to ADHD. As this impacts on their engagement with services, age‐appropriate psycho‐educational initiatives delivered in nonstigmatising settings need to be developed.
- Interventions to improve the access of children at risk of ADHD to appropriate care have targeted the knowledge of professionals or aimed to enhance the communication between key adults; the effectiveness of those interventions was limited and their long‐term benefit has not been evaluated.
- There is a need for clinical guidelines to be appropriately implemented.

## Supporting information


**Appendix S1.** Literature review search terms.
**Appendix S2.** Excluded studies table.
