# Supplementary material for: Practitioner Review: Pathways to care for ADHD – a systematic review of barriers and facilitators
Source: J Child Psychol Psychiatry. 2015 Feb 23;56(6):598–617. doi: 10.1111/jcpp.12398 (PMC5008177; doi:10.1111/jcpp.12398)
Supplement: Supplementary file 1 — Appendix S1. Literature review search terms. Appendix S2. Excluded studies table. [file JCPP-56-598-s001.docx]

*Online supplementary material for -* ***Pathways to Care for ADHD: A Systematic Review of Barriers and Facilitators; by Wright et al***

[Appendix S1. Literature Review Search Terms](#AppS1)

[Appendix S2. Excluded Studies Table](#AppS2)

Appendix S1. Literature Review Search Terms

PUBMED search strategy for Medline

| *1* | Search attention and deficit* |
| --- | --- |
| *2* | Search minimal and dysfunction* |
| *3* | Search attention and hyperactivit* |
| *4* | Search Hyperkinetic and Syndrome* |
| *5* | Search "Attention Deficit and Disruptive Behavior Disorders"[Mesh] OR "Attention Deficit Disorder with Hyperactivity"[Mesh] |
| *6* | Search #1 or #2 or #3 or #4 or #5 |
| *7* | Search health services accessibility/ or healthcare disparities/ |
| *8* | Search "health services accessibility"[Mesh] OR " Delivery of Health Care "[Mesh] |
| *9* | Search (access* or advoca* or barrier* carer* or communication* or information* or uptake or utilization or need* or provision or consent* or help seeking or help-seeking) |
| *10* | Search #7 or #8 or #9 |
| *11* | *Search #6 and #10* |

# EMBASE search strategy (www.embase.com)

| *1* | exp attention deficit disorder |
| --- | --- |
| *2* | (attention adj5 deficit).mp. |
| *3* | (adhd or adhkd or addh or adhs).tw. |
| *4* | hyperactiv$.mp. |
| *5* | exp hyperkinesia |
| *6* | hyperkine$.mp. |
| *7* | (attention and hyperactivi$).mp. |
| *8* | (minimal and dysfunction$).mp. |
| *9* | **1 or 2 or 3 or 4 or 5 or 6 or 7 or 8** |
| *10* | (access or advoc$ or barrier$ or carer$ or communicati$).mp. |
| *11* | (information$ or uptake or utilization or need$ or provision).mp. |
| *12* | (consent or help-seeking).mp. |
| *13* | (help adj1 seeking).mp. |
| *14* | health care delivery/ |
| *15* | **10 or 11 or 12 or 13 or 14** |
| *16* | *9 and 15* |

# Search strategy for the “American Psychological Association” databases (APA PsycNET - http://psycnet.apa.org/)

1. **PsycINFO**: bibliographic database of scholarly literature in the psychological, social, behavioral, and health sciences. PsycINFO covers journals, books, reviews, and dissertations.
2. **PsycBOOKS**: full-text database of APA books, classic books, and entries from the Encyclopedia of Psychology.
3. **PsycCRITIQUES**: full-text database of book and film reviews.
4. **PsycARTICLES**: full-text database of journals published by APA and other publishers in subject areas such as applied psychology, health, theory, research, social/personality, and more.
5. **PsycEXTRA**: a gray literature database. Gray literature is material written for professionals and disseminated outside of peer-reviewed journals. Documents include newsletters, magazines, newspapers, technical and annual reports, government reports, consumer brochures, and more.

| *1* | hyperactivity AND ‘access care’ |
| --- | --- |
| *2* | ‘attention deficit disorder’ AND ‘access care’ |
| *3* | *1 and 2* |

# Cochrane Library (Issue 6, 2010) search strategy

| *1* | MeSH descriptor Attention Deficit and Disruptive Behavior Disorders explode all trees |
| --- | --- |
| *2* | MeSH descriptor Attention Deficit Disorder with Hyperactivity explode all trees |
| *3* | (Hyperkinetic and Syndrome*) or (attention and hyperactivit*) or (minimal and dysfunction*) or (attention and deficit*) |
| *4* | (#1 OR #2 OR #3) |
| *5* | ((access* or advoca* or barrier* carer* or communication* or information* or uptake or utilization or need* or provision or consent* or (help near seeking) or help-seeking)) |
| *6* | MeSH descriptor Health Services Accessibility explode all trees |
| *7* | MeSH descriptor Delivery of Health Care explode all trees |
| *8* | health services accessibility/ or healthcare disparities/ |
| *9* | (#5 OR #6 OR #7 OR #8) |
| *10* | (#4 AND #9) |

Cochrane Library® is composed of five databases. This search retrieved 2125 references in CENTRAL, 481 in the CDSR, 96 references in DARE, 63 references in EES, and 25 references in the HTA databases.

# Additional searches

# TRIP database

Trip database (<http://www.tripdatabase.com/>) was searched using the following terms "[(‘attention deficit disorder’ or hyperactivity) AND ‘access care barrier’]"Controlled Trials Register and 15 relevant references were retrieved.

# World Bank Documents & Reports

World Bank Documents & Reports (<http://www-wds.worldbank.org/>) was searched using the following terms "[(‘attention deficit disorder’ or hyperactivity) AND ‘access care barrier’]" and three relevant references were retrieved

# Organization for Economic Co-operation and Development (OECD)

OECD Publications & Documents ([www.oecd.org](http://www.oecd.org)) was searched using the following terms "[(‘attention deficit disorder’ or hyperactivity) AND ‘access care barrier’]" and four relevant references were retrieved.

# Health Development Agency

Health Development Agency (<http://www.hda-online.org.uk/>) was searched using the term “attention deficit”, and no relevant references were retrieved.

# National Primary Care Research and Development Centre

National Primary Care Research and Development Centre ([www.npcrdc.man.ac.uk](http://www.npcrdc.man.ac.uk)) was searched using the term “attention deficit”, and no relevant references were retrieved.

# Children’s Society

Children’s Society (<http://www.the-childrens-society.org.uk/>) was searched using the term “attention deficit”, and no relevant references were retrieved.

Appendix S2. Excluded Studies Table

| **Article Details** | **Reason for Exclusion** |
| --- | --- |
| Akram G et al (2009) ADHD and the role of medication: Knowledge and perceptions of qualified and student teachers. European Journal of Special Needs Education 24 (2): 423-436 | Does not examine access to care for ADHD |
| Allen & Glavina (2004) An audit of an ADHD assessment clinic in light of NICE guidelines. Clinical Governance 9 (3): 167-171 | Not an empirical study |
| Apres 10 ans d'activite, HyperSupers-TDAH France dresse un premier bilan de ses actions au travers des resultats d'une enquete menee durant l'ete 2011 aupres des familles adherentes." ANAE - Approche Neuropsychologique des Apprentissages chez l'Enfant 23(114): 358-364 | Not written in English |
| Arcia A & Fernandez MC (2003) From awareness to acknowledgement. Journal of Attention Disorders 6 (4): 163-175 | Less than 50% of the sample has ADHD characteristics |
| Arcia E et al (2004) Modes of entry into services for young children with disruptive behaviours. Qualitative Health Research 14 (9): 1211-1226 | Less than 50% of the sample has ADHD characteristics |
| Arcia E & Fernandez MC (2003) Presenting problems and assigned diagnoses among young Latino children with disruptive behaviours. Journal of Attention Disorders 6 (4): 177-185 | Less than 50% of the sample has ADHD characteristics |
| Arcia E et al (2004) Latina mothers’ stances on stimulant medication: complexity, conflict and compromise. Journal of Developmental and Behavioural Pediatrics 25 (5): 311-317 | Less than 50% of the sample has ADHD characteristics |
| Atkins MS et al (2003) Towards a new model for promoting urban children’s mental health: Accessible, effective and sustainable school-based mental health services. School Psychology Review 32 (4): 503 -514 | Less than 50% of the sample has ADHD characteristics |
| Bachman JA et al (2000) A world wide web based health resource. Survey of Missouri school nurses to determine priority health information resources for SchoolhealthLink. Journal of School Nursing 16 (1): 28-33 | No extractable data related to ADHD or its symptoms |
| Bailey RK et al (2010) Attention-deficit/hyperactivity disorder in African American youth. Current Psychiatric Reports 12 (5): 396-402 | Not an empirical study |
| Baldwin L (2002) Keep taking the tablets? Evidence based approaches to ADHD. Part 2: Audit and changing practice. Paediatric Nursing 14 (4): 34-37 | Does not examine access to care for ADHD |
| Baldwin, S. (2000) Impact evaluation of a mass media public education campaign on clinic service provision for minors diagnosed with ADHD/ADD: Audit survey of 100 index families International Journal of Risk and Safety in Medicine 13 (4): 203-219 | Does not examine access to care for ADHD |
| Barbaresi, W. J. & Olsen, R. D. (1998) An ADHD Educational Intervention for Elementary Schoolteachers: A Pilot Study, Developmental and Behavioural Paediatrics 19 (2): 94-100 | Does not examine access to care for ADHD |
| Barnard-Brak L et al (2009) Examining parental non-response to stimulant treatment questions according to ethnicity. Journal of Child and Adolescent Psychopharmacology 19 (3): 301-304 | Does not examine access to care for ADHD |
| Beckle B (2004) Knowledge and attitudes about attention-deficit hyperactivity disorder (ADHD): A comparison between practicing teachers and undergraduate education students. Journal of Attention Disorders 7 (3): 151-161 | Does not examine access to care for ADHD |
| Bennett DS et al (1996) Parent acceptability and feasibility of ADHD interventions: assessment, correlates and predictive validity. Journal of Pediatric Psychology 21 (5): 643-657 | Sample comprises children with an existing diagnosis of ADHD |
| Berger I et al (2008) Attitudes toward Attention Deficit Hyperactivity Disorder (ADHD) treatment: parents and children’s perspectives. Journal of Child Neurology 23 (9): 1036-1042 | Sample comprises children with an existing diagnosis of ADHD |
| Berger-Jenkins E et al (2012) Parent medication concerns predict under-utilization of mental health services for minority children with ADHD. Clinical Pediatrics 51 (1): 65-76 | Sample comprises children with an existing diagnosis of ADHD |
| Bhatara V et al. (2003) Web-based classroom data collection in ADHD: A best practices case study. South Dakota Journal of Medicine 56 (9): 385-389 | Does not examine access to care for ADHD |
| Bokhari FAS & Schneider H (2011) School accountability laws and the consumption of psychostimulants. Journal of Health Economics 30 (2): 355-372 | Not an empirical study |
| Bokhari FAS and Schneider H (2011) School accountability laws and the consumption of psychostimulants. Journal of Health Economics 30 (2): 355-372 | Sample comprises children with an existing diagnosis of ADHD |
| Breuer D & Dopfner M (2006) [Attention deficit/hyperactivity disorders among three to six year olds treated in medical practices – a national survey.] Zeitschrift fur Kinder und Jugendpsychiatrie und Psychotherapie | Not written in English |
| Brewis A & Schmidt KL (2003) Gender variation in the identification of Mexican children’s psychiatric symptoms. Medical Anthropology Questions 17 (3): 376-393 | Does not examine access to care for ADHD |
| Brinkman et al (2009) Parental angst making and decisions about treatment of ADHD. Pediatrics 124 (2): 580-589 | No extractable data related to ADHD or its symptoms |
| Brinkman, W. B., J. Hartl, et al. (2011). "Physicians' shared decision-making behaviors in attention-deficit/ hyperactivity disorder care." Archives of Pediatrics and Adolescent Medicine 165(11): 1013-101 | Does not examine access to care for ADHD |
| Brinkman, W. B., S. N. Sherman, et al. (2012). "In their own words: adolescent views on ADHD and their evolving role managing medication." Acad Pediatr 12(1): 53-61. | Does not examine access to care for ADHD |
| Brook, U et al (2000) Attitude and knowledge of attention deficit hyperactivity disorder and learning disability among high school teachers, Patient Education and Counselling 40 (3): 247 -252 | Does not examine access to care for ADHD |
| Brown JD et al (2007) Receiving advice about child mental health from a primary care provider: African American and Hispanic parent attitudes. Medical Care 45 (11): 1076-1082 | No extractable data related to ADHD or its symptoms |
| Brown, J. J., J. L. Hertzer, et al. (2011). "Assessment of core competencies in childhood attention-deficit/ hyperactivity disorder practice." Journal of Child and Adolescent Psychopharmacology 21(1): 33-41 | Does not examine access to care for ADHD |
| Brownell & Yogendran (2001) ADHD in Manitoba children: Medical diagnosis and psycho-stimulant treatment rates. Canadian Journal of Psychiatry 46 (3): 264-272 | Sample comprises children with an existing diagnosis of ADHD |
| Burgess IC (2002) Service innovations: Attention-deficit hyperactivity disorder: development of a multi-professional integrated care pathway. Psychiatric Bulletin 26 (4): 148-151 | Not an empirical study |
| Bussing et al (2007) Cultural variations in parental health beliefs, knowledge and information sources relation to ADHD. Journal of Family Issues 28 (3): 291-318 | Does not examine access to care for ADHD |
| Bussing R & Gary FA (2001) Practice Guidelines and Parental ADHD Treatment Evaluations: Friends or Foes? Harvard Rev Psychiatry 9 (5): 223-233 | Does not examine access to care for ADHD |
| Bussing R et al (1998) Explanatory models of ADHD: Do they differ by ethnicity, child gender, or treatment status? Journal of Emotional and Behavioral Disorders 6 (4): 233-242 | Does not examine access to care for ADHD |
| Bussing R et al (1998) Knowledge and information about ADHD: Evidence of cultural differences among African-American and white parents. Social Science and Medicine 46 (7): 919-928 | Does not examine access to care for ADHD |
| Bussing R et al (2003) Agreement between CASA parent reports and provider records of Children’s ADHD services. Journal of Behavioural Health Services Research 30 (4): 462-469 | Does not examine access to care for ADHD |
| Bussing R et al (2003) Parental explanatory models of ADHD Gender and cultural variations. Social Psychiatry and Psychiatric Epidemiology 38 (10): 563-575 | Does not examine access to care for ADHD |
| Bussing R et al (2005) Use and persistence of pharmacotherapy for elementary school students with attention-deficit/hyperactivity disorder. Journal of Child and Adolescent Psychopharmacology 15 (1): 78-87 | Does not examine access to care for ADHD |
| Bussing R, et al (1998) Variations in ADHD treatment among special education students. Journal of the American Academy of Child and Adolescent Psychiatry 37 (9): 968-976 | Does not examine access to care for ADHD |
| Chan E et al (2002) Health care use and costs for children with attention deficit/hyperactivity disorder: National estimates from the medical expenditure panel survey. Archives of Pediatric Adolescent Medicine 156 (5): 504-511 | Less than 50% of the sample has ADHD characteristics |
| Chen CY et al (2009) Determinants of initial pharmacological treatment for youths with attention-deficit/hyperactivity disorder. Journal of Child and Adolescent Psychopharmacology 19 (2): 187-195 | Less than 50% of the sample has ADHD characteristics |
| Chu S (2003) Occupational therapy for children with ADHD: A survey on the level of involvement and training needs of therapists. British Journal of Occupational Therapy 66 (5): 01 | Does not examine access to care for ADHD |
| Co et al (2010) Electronic health record decision support and quality care for children with ADHD. Pediatrics 2: 239-246 | Does not examine access to care for ADHD |
| Coker et al (2009) Racial/ethnic disparities in the mental health care utilization of fifth grade children. Academy of Pediatrics 9 (2): 89-96 | Does not examine access to care for ADHD |
| Coleman D et al (2009) Children’s beliefs about causes of childhood depression and ADHD: A study of stigmatisation. Psychiatric Services 60 (7): 950-957 | Does not examine access to care for ADHD |
| Connor DF et al (2006) Targeted child psychiatric services: A new model of pediatric primary clinician-child psychiatry collaborative care. Clinical Pediatrics 45 (5): 423-434 | Does not examine access to care for ADHD |
| Copeland L et al (1987) Pediatricians’ reported practices in the assessment and treatment of Attention Deficit Disorders. Developmental and Behavioural Pediatrics 8 (4): 191- 197 | Does not examine access to care for ADHD |
| Corcoran J & Dattalo P (2006) Patient involvement in treatment for ADHD: A meta-analysis of the published studies. Research on Social Work Practice 16(6): 561-570 | Not an empirical study |
| Corkum P et al (1999) Parental knowledge of ADHD and opinions of treatment options: Impact on enrolment and adherence to a 12 month treatment trial. Canadian Journal of Psychiatry 44 (10): 1043-1048 | Does not examine access to care for ADHD |
| Cornelius JR et al (2001) Correlates of mental health service utilisation and unmet need among a sample of male adolescents. Addictive Behaviours 26 (1): 11-19 | Less than 50% of the sample has ADHD characteristics |
| Couture C et al (2003) Comparison of Quebec and British teachers beliefs about, training in and experience with attention deficit hyperactivity disorder. Emotional and Behavioural Difficulties 8 (4) 284-302 | Does not examine access to care for ADHD |
| Daly M et al (2006) Assessment and diagnosis of ADHD by familyphysicians. Minnesota Medicine 89 (3): 40-43 | Does not examine access to care for ADHD |
| Damico JS & Augustine LE (1995) Social considerations in the labelling of students as attention deficit hyperactivity disordered. Seminars in Speech and Language 16 (4): 259-273 | Does not examine access to care for ADHD |
| Damodaran J et al (2012) Preliminary evaluation after setting up a post-assessment attention deficit hyperactivity disorder clinic in a community setting. Irish Journal of Medical Science 181 (1): 147-150 | Sample comprises children with an existing diagnosis of ADHD |
| De Ramirez R & Shapiro ES (1998) Teacher ratings of ADHD symptoms in Hispanic children. Journal of Psychopathology and Behavioural Assessment 20 (4): 275-293 | Does not examine access to care for ADHD |
| Demidovich, M. et al. (2011). "Medication refusal in children with oppositional defiant disorder or conduct disorder and comorbid attention-deficit/hyperactivity disorder: medication history and clinical correlates." Journal of Child and Adolescent Psychopharmacol 21(1): 57-66 | Does not examine access to care for ADHD |
| Dennis T et al (2008) ADHD: Parents and professional perceptions. Community Practitioner 81 (3): 24-28 | Does not examine access to care for ADHD |
| DosReis S et al(2003) Parental perceptions and satisfaction with stimulant medication for attention- deficit hyperactivity disorder. Journal of Dvelopmental and Behavioural Pediatrics | Does not examine access to care for ADHD |
| DosReis S et al (2004) Multimodal treatment for ADHD among youths in three Medicaid subgroups: Disabled, foster care and low income. Psychiatric Services 55 (9): 1041-1048 | Sample comprises children with an existing diagnosis of ADHD |
| DosReis S et al (2006) Attitudes about stimulant medication for attention-deficit/hyperactivity disorder among African American families in an inner city community. Journal of Behavioural Health Services Research 33 (4): 423-430 | Sample comprises children with an existing diagnosis of ADHD |
| dosReis S et al (2007) Coming to terms with ADHD: how urban African-American families come to seek care for their children. Psychiatric Services 58 (5): 636-641 | Sample comprises children with an existing diagnosis of ADHD |
| DosReis S et al (2009) The meaning of attention deficit hyperactivity disorder medication and parents initiation and continuity of treatment for their child. Journal of Child and Adolescent Psychopharmacology 19 (4): 377 - 383 | Sample comprises children with an existing diagnosis of ADHD |
| Dreyer AS et al (2010) Parental adherence to clinical recommendations in an ADHD evaluation clinic. Journal of Clinical Psychology 66: 1-20 | Does not examine access to care for ADHD |
| Eiraldi R & Diaz Y (2010) Use of treatment services for attention-deficit/hyperactivity disorder in Latino children. Current Psychiatric Reports 12 (5): 403-408 | Not an empirical study |
| Epstein et al (2011) Use of an internet portal to improve community based pediatric care: A cluster randomised control trial. Pediatrics 128 (5) e1201-1208 | Does not examine access to care for ADHD |
| Epstein JN et al (2007) Improving ADHD treatment outcomes through use of a collaborative consultation treatment service by community based paediatricians: A cluster randomised trial. Archives of Pediatric Adolescent Medicine 161 (9): 835-840 | Does not examine access to care for ADHD |
| Epstein JN et al (2008) Community-wide intervention to improve the ADHD assessment and treatment practices of community physicians. Pediatrics 122 (1): 19-27 | Does not examine access to care for ADHD |
| Ezpeleta L et al (2009) Use of mental health services and unmet needs for socially at-risk Spanish children and adolescents Epidemiologia e Psichiatria sociale 18 (2): 147-153 | Does not examine access to care for ADHD |
| Faber A et al (2010) Co-morbidity and patterns of care in stimulant treated children with ADHD in the Netherlands. European Child and Adolescent Psychiatry 19 (2): 159-166 | Does not examine access to care for ADHD |
| Fanton JH et al (2008) Preschool parent-pediatrician consultants and predictive referral pattern for problematic behaviours Journal of Developmental and Behavioural Pediatrics 29 (6): 475-482 | Less than 50% of the sample has ADHD characteristics |
| Fiks, A. G., C. C. Hughes, et al. (2011). "Contrasting parents' and pediatricians' perspectives on shared decision-making in ADHD." Pediatrics 127(1): e188-e196 | Does not examine access to care for ADHD |
| Fine S et al (1999) Parent satisfaction with receiving information in an ADHD clinic. Psychiatric Bulletin 23 (5): 304-305 | Does not examine access to care for ADHD |
| Flannagan D & Pillow DR (2002) Perceptions and communications about ADHD and ODD behaviours in children with combined type ADHD. Childrens Health Care 31 (3): 223-236 | Does not examine access to care for ADHD |
| Flowers A & McDougle L (2010) In search of an ADHD screening tool for African American children. Journal of National Medical Association 102 (5): 372-374 | Not an empirical study |
| Forrester, M., K. Forsyth, et al. (2011). "Transition to adult services for young people with ADHD: The views of a range of professionals." Child and Adolescent Mental Health 16: 15 | Does not examine access to care for ADHD |
| Foy JM and Earls MF (2005) A process for developing community consensus regarding the diagnosis and management of ADHD. Pediatrics 115 (1): e97-104 | Does not examine access to care for ADHD |
| Fremont WP et al (2008) Comfort level of paediatricians and family medicine physicians diagnosing and treating child and adolescent psychiatric disorders. International Journal of Psychiatry Medicine 38 (2): 153-168 | Does not examine access to care for ADHD |
| Frisch, L. et al (2003) Kansas school nurses’ knowledge and opinions about ADHD stimulant medication therapy, Journal of School Health 73 (3): 127-128 | Does not examine access to care for ADHD |
| Fulton BD et al (2009) National variation of ADHD diagnostic prevalence and medication use: health care providers and education policies. Psychiatric Services 60 (8): 1075-1083 | Sample comprises children with an existing diagnosis of ADHD |
| Gardner W et al (2002) Child sex differences in primary care clinicians’ mental health care of children and adolescents. Archives of Paediatric and Adolescent Medicine 156 (5): 454-459 | Does not examine access to care for ADHD |
| Getin-Vergnaud, C. and K. G. Angenon-Delerue (2011). "After 10 years in operation, "HyperSupers TDAH -France" carries out a first assessment of its supporting actions for families with ADHD children through the results of a survey conducted in summer 2011 among member families. [French] ANAE - Approche Neuropsychologique des Apprentissages chez l'Enfant 23 (114): 358-364 | Not written in English |
| Ghanizadeh A (2007) Educating and counselling of parents of children with ADHD. Patient Education and Counselling 68 (1): 23-28 | Does not examine access to care for ADHD |
| Ghanizadeh A (2008) Knowledge of pharmacists regarding Ritalin and ADHD and their attitude towards the use of Ritalin to treat ADHD. International Journal of Clinical Pharmacology and Therapy 46 (2): 84-88 | Does not examine access to care for ADHD |
| Ghanizadeh A et al (2009) Disclosure of attention deficit hyperactivity disorder and its effect on rejection of students by teachers. Iranian Journal of Medical Sciences 34 (4): 259-264 | Does not examine access to care for ADHD |
| Ghanizadeh, A et al (2006) Knowledge and attitudes towards attention deficit hyperactivity disorder among elementary school teachers, Patient Education and Counselling, 63 (1-2): 84-88 | Does not examine access to care for ADHD |
| Ghanizadeh, A. & Zarei, N. (2010) Are GPs adequately equipped with the knowledge for educating and counselling of families with ADHD children?, BMC Family Practice, 11 (5) | Does not examine access to care for ADHD |
| Gomes M et al (2007) Knowledge about ADHD in Brazil. J Bras Psiquiatr 56 (2): 94-101 | Not written in English |
| Guevara J et al (2001) Utilization and cost of health care services for children with attention-deficit/hyperactivity disorder. Pediatrics 108 (1): 71-78 | Does not examine access to care for ADHD |
| Guevara JP et al (2005) Fragmented care for inner-city minority children with ADHD. International Journal of Psychiatry and Medicine 116 (4): e512-517 | Does not examine access to care for ADHD |
| Hakkaart van Roijen L et al (2007) Societal costs and quality of life of children suffering from attention deficit hyperactivity disorder. European Child and Adolescent Psychiatry 16 (5): 316-326 | Does not examine access to care for ADHD |
| Handler MW & DuPaul G (2005) Assessment of ADHD: Differences across psychology specialty areas. Journal of Attention Disorders 9 402-412 | Does not examine access to care for ADHD |
| Harborne A et al (2004) Making sense of ADHD: A battle for understanding? Parents’ views of their children being diagnosed with ADHD. Clinical Child Psychology and Psychiatry 9 (3): 327-339 | Does not examine access to care for ADHD |
| Harpur RA et al (2008) The ADHD medication related attitudes of patients and their parents. Journal of Child and Adolescent Psychopharmacology 18 (5): 461-473 | Sample comprises children with an existing diagnosis of ADHD |
| Harvey JM et al (2005) Teachers perceptions of the incidence and management of ADHD. Applied Neuropsychology 12 (2): 120-127 | Does not examine access to care for ADHD |
| Heiervang E et al (2007) Psychiatric disorders in Norwegian 8 to 10 year olds: An epidemiological survey of prevalence, risk factors and service user. Journal of the American Academy of Child and Adolescent Psychiatry 46 (4): 438-447 | Less than 50% of the sample has ADHD characteristics |
| Heikkinen A et al (2002) Child psychiatric skills in primary healthcare: Self-evaluation of Finnish health centre doctors. Child Care Health Development 28 (2): 131-137 | Does not examine access to care for ADHD |
| Hellwig-Brida S et al (2009) [The parent questionnaire “ADHD-knowledge and motivation for treatment” – development and first results]. Z Kinder Jugendpsychiatry Psychotherapie 37 (5): 441-449 | Not written in English |
| Heneghan A et al (2008) Pediatricians’ role in providing mental health care for children and adolescents: Do paediatricians and child and adolescent psychiatrists agree? Journal of Developmental and Behavioural Pediatrics 29 (4): 262-269 | No extractable data related to ADHD or its symptoms |
| Hervey-Junmper H et al (2006) Deficits in diagnosis, treatment and continuity of care in African-American children and adolescents with ADHD. Journal of the National Medical Association 98 (2): 233-238 | Does not examine access to care for ADHD |
| Hinshaw, S. P., R. M. Scheffler, et al. (2011). "International variation in treatment procedures for ADHD: Social context and recent trends." Psychiatric Services 62(5): 459-464 | No extractable data related to ADHD or its symptoms |
| Hirfanoglu T et al (2008) A study of perceptions, attitudes and level of knowledge among paediatricians towards attention-deficit/hyperactivity disorder. Turkish Journal of Pediatrics 50 (2): 160-166 | Does not examine access to care for ADHD |
| Hoagwood, K et al (2000) Medication management of stimulants in pediatric practice settings: a national perspective, Developmental and behavioural Paediatrics, 21 (5): 322-331 | Less than 50% of the sample has ADHD characteristics |
| Holling H et al (2007) [Behavioural problems in children and adolescents. First results from the German health interview and examination survey for children and adolescents (KiGGS)]. Bundesgesundheitsblatt Gesundheitsforschung Gesundheitsschutz 50 (5-6): 784-793 | Not written in English |
| Hugtenburg JG et al (2004) Treatment of children with methylphenidate: Is there compliance with the instructions for use? Tijdschift voor Psychiatrie 46 (1): 7-14 | Not written in English |
| Hundevadt L (2000) [Hyperactive children meeting the health service system] Tidsskr Nor Laegeforen 120 (5): 584-587 | Not written in English |
| Ibrahim SR (2002) Rates of adherence to pharmacological treatment among children and adolescents with ADHD. Human Psychopharmacology 17 (5): 225-231 | Sample comprises children with an existing diagnosis of ADHD |
| Ishizaki Y et al. (2005) Research on promotion of management of children with psychosomatic disorders in Japan. Pediatric International 47 (3): 352-357 | Does not examine access to care for ADHD |
| Jackson, D.& Peters, K. (2008) Use of drug therapy in children with attention deficit hyperactivity disorder (ADHD): maternal views and experiences, Journal of Clinical Nursing 17 (20): 2725-2732 | Does not examine access to care for ADHD |
| Jans et al (2009) Does the treatment of maternal ADHD enhance the efficacy of a behavioural parent training for the treatment of their childrens ADHD? Study protocol of a randomised controlled multicentred trial. Attention Deficit and Hyperactivity Disorders 1 (1): 33-45 | Not an empirical study |
| Jarque-Fernández, S et al (2007) Conocimientos, concepciones erróneas y lagunas de los maestros sobre el trastorno por déficit de atención con hiperactividad (Teachers’ knowledge, misconceptions, and lacks concerning Attention Deficit Hyperactivity Disorder), Psicothema, 19 (4): 585-590 | Not written in English |
| Jawaid, A. et al (2008) Knowledge of primary paediatric care providers regarding attention deficit hyperactivity disorder and learning disorder: a study from Pakistan, Singapore Medical Journal 49 (12): 985-993 | Does not examine access to care for ADHD |
| Johnston C et al (2005), Treatment choices and experiences in attention deficit and hyperactivity disorder: relations to parents’ belief and attributions. Child care Health and Development 31 (6): 669-677 | Sample comprises children with an existing diagnosis of ADHD |
| Johnston C et al (2008) Acceptability of behavioural and pharmacological treatments for ADHD: Relations to child and parent characteristics. Behaviour Therapy 39 (1): 22-32 | Sample comprises children with an existing diagnosis of ADHD |
| Johnston, C. et al (2011)The Effects of Instructions on Mothers’ Ratings of Child Attention-Deficit/Hyperactivity Disorder Symptoms. Journal of Abnormal Child Psychology 39 (8): 1099-1110 | Sample comprises children with an existing diagnosis of ADHD |
| Kasten E (1992) Educators knowledge and attitudes regarding stimulants in the treatment of ADHD. Developmental and Behavioural Paediatrics 13 (3): 215-219 | Does not examine access to care for ADHD |
| Kendall J et al (2005) Service needs of families with children with ADHD. Journal of Family Nursing 11 (3): 264-288 | Sample comprises children with an existing diagnosis of ADHD |
| Kildea S et al (2011) Making sense of ADHD in practice: A stakeholder review. Clinical Child Psychology and Psychiatry 16 (4): 599-619 | Sample comprises children with an existing diagnosis of ADHD |
| Killic et al (2007) Sociodemographic and clinical factors associated with compliance to methylphenidate treatment in children with ADHD. Turk Psikiyatri Derg 18 (3): 207-213 | Not written in English |
| Klasen H & Goodman R (2000) Parents and GPs at cross-purposes over hyperactivity: A qualitative study of possible barriers to treatment. British Journal of General Practice 50: 199-202 | Sample comprises children with an existing diagnosis of ADHD |
| Klasen H (2000) A name, What’s in a name? The medicalisation of Hyperactivity revisted. Harvard Review of Psychiatry 7 (6): 334-344 | Sample comprises children with an existing diagnosis of ADHD |
| Knapp CA. et al (2011) Factors Associated with a Medical Home Among Children with Attention-Deficit Hyperactivity Disorder. Maternal and Child Health. Maternal Child Health Journal 2011/12/07 | Sample comprises children with an existing diagnosis of ADHD |
| Knipp (2006) Teens perceptions about ADHD and medications. Journal of School Nursing 22 (2): 120-125 | Does not examine access to care for ADHD |
| Koro-Ljungberg M et al (2008) African-American teenagers’ stories of attention deficit/hyperactive disorder. Journal of Child and Family Studies 17 (4): 467-485 | Does not examine access to care for ADHD |
| Kotowycz N et al (2005) Assessing the standard of care for child and adolescent ADHD in Elgin County, Ontario: A pilot study. Canadian Journal Rural Medicine 10 (3): 149-154 | Does not examine access to care for ADHD |
| Krain AL et al (2005) The role of treatment acceptability in the initiation of treatment for ADHD. Journal of Attention Disorders 9 (2): 425-434 | Sample comprises children with an existing diagnosis of ADHD |
| Kriz S & Thomsen PH (2009) Doubling of the capacity of child psychiatric services in a region of south western Norway. Nordic Journal of Psychiatry 63 (4): 322-330 | No extractable data related to ADHD or its symptoms |
| Kwasman A et al (1995) Pediatricians’ knowledge and attitudes concerning diagnosis and treatment of ADHD. Archives of Paediatric and Adolescent Medicine 149 (11): 1211-1216 | Does not examine access to care for ADHD |
| Kwasman A et al (2004) School nurses knowledge and beliefs about the management of children with ADD. Journal of School Nursing 20 (1): 22-28 | Does not examine access to care for ADHD |
| Langberg JM et al (2009) Interventions to promote evidence-based care of children with ADHD in primary care settings. Expert Review of Neurotherapeutics xx (4): 477-487 | Not an empirical study |
| Lanham JS (2006) The evaluation of Attention Deficit/Hyperactivity Disorder in family medicine residency programs. Southern Medical Journal 99 (8) 803-805 | Does not examine access to care for ADHD |
| Lannon C et al (2007) Partnerships for quality project: Closing the gap in care of children with ADHD. Joint Commission Journal on Quality and Patient Safety 33 (12Suppl): 66-74 | Does not examine access to care for ADHD |
| Lavigne JV et al (2011) Computer assisted management of attention-deficit/hyperactivity disorder. Pediatrics 128 (1) e46-e53 | Sample comprises children with an existing diagnosis of ADHD |
| Lavigne JV et al (2012) Can parent reports serve as a proxy for teacher rating in medication management of ADHD? Journal of Developmental and Behavioural Pediatrics 2012/03/01 | Does not examine access to care for ADHD |
| Leggett, C. and E. Hotham (2011). "Treatment experiences of children and adolescents with attention-deficit/ hyperactivity disorder." Journal of Paediatrics and Child Health 47(8): 512-517. | Does not examine access to care for ADHD |
| Lesesne CA et al (2003) ADHD in school-aged children: Association with maternal mental health and use of health care resources. Pediatrics 111 (5): 1232-1237 | Sample comprises children with an existing diagnosis of ADHD |
| Leslie DL et al(2001) Patterns of mental health utilisation and costs among children in a privately insured population. Health Services Research 36 (1): 113-127 | Less than 50% of the sample has ADHD characteristics |
| Leslie et al (2007) Investigating ADHD treatment trajectories: listening to families stories about medication use. Journal of Developmental and Behavioural Pediatrics 28 (3): 179-188 | Does not examine access to care for ADHD |
| Leslie LK et al (2004) Implementing the American Academy of Pediatrics ADHD diagnostic guidelines in primary care settings. Pediatrics. 114 (1): 129-140 | Does not examine access to care for ADHD |
| Leslie, L. K., A. M. Rodday, et al. (2012). "Cardiac screening prior to stimulant treatment of ADHD: a survey of US-based pediatricians." Pediatrics 129(2): 222-230 | Does not examine access to care for ADHD |
| Lian WB et al (2008) Pre-school teachers knowledge attitudes and practices on childhood developmental and behavioural disorders in Singapore. Journal of Pediatric and Child Health 44 (4): 187-194 | Does not examine access to care for ADHD |
| Lin YF & Chung HH (2002) Parenting stress and parents’ willingness to accept treatment in relation to behavioural problems of children with attention-deficit hyperactivity disorder. Journal of Nursing Research 10 (1): 43-56 | Does not examine access to care for ADHD |
| Little M & McLennan JD (2010) Teacher perceived mental and learning problems of children referred to a school mental health service. Journal of the Canadian Academy of Child and Adolescent Psychiatry 19 (2): 94-99 | Does not examine access to care for ADHD |
| Lobar S et al (1999) Parents, physicians and nurse practitioners perceptions of behaviours associated with ADHD. Journal of the American Academy of Nurse Practitioners 11(6) | Sample comprises children with an existing diagnosis of ADHD |
| Louw C et al (2009) GP familiarity, attitudes and practices with regard to ADHD in children and adults. South African Family Practice 51 (2): 152-157 | Does not examine access to care for ADHD |
| MacNaughton KL & Rodrigue JR (2001) Predicting adherence to recommendations by parents of clinic-referred children. Journal of Consulting and Clinical Psychology 69 (2): 262-270 | Less than 50% of the sample has ADHD characteristics |
| Mah JW & Johnston C (2007) Cultural variations in mothers’ attributions: Influence of child attention-deficit/hyperactivity disorder. Child Psychiatry and Human Development 38 (2): 135-153 | Does not examine access to care for ADHD |
| Marcer H et al (2008) ADHD and transition to adult services: The experience of community paediatricians. Child care Health Development 34 (5): 564-566 | Does not examine access to care for ADHD |
| Marcus SC & Durkin M (2011) Stimulant adherence and academic performance in urban youth with attention-deficit/hyperactivity disorder. Journal of the American Academy of Child and Adolescent Psychiatry 50 (5): 480-489 | Sample comprises children with an existing diagnosis of ADHD |
| Martinussen R et al (2011) Teachers’ reported use of instructional and behaviour management practices for students with behaviour problems: Relationship to role and level of training in ADHD. Child and Youth Care Forum 40 (3): 193-210 | Does not examine access to care for ADHD |
| Mattingly, G., C. B. Surman, et al. (2011). "Improving Communication in ADHD Care: Results from In-office Linguistic Research." CNS Spectrums 2011/05/04 | Does not examine access to care for ADHD |
| McGonnell M et al (2009) Doing it right: An interdisciplinary model for the diagnosis of ADHD. Journal of the Canadian Academy of Child and Adolescent Psychiatry 18 (4): 283-286 | Does not examine access to care for ADHD |
| McIntyre, R. and E. Hennessy (2012). "'He's just enthusiastic. Is that such a bad thing?' Experiences of parents of children with Attention Deficit Hyperactivity Disorder." Emotional and Behavioural Difficulties 17(1): 65-82 | Sample comprises children with an existing diagnosis of ADHD |
| McKenzie I & Wurr C (2004) Diagnosing and treating attentional difficulties: A nationwide survey. Archives of Disorders in Children 89 (10): 913-916 | Does not examine access to care for ADHD |
| McLeod JD et al (2007) Public knowledge, beliefs and treatment preferences concerning ADHD. Psychiatric Services 58 (5): 626-631 | Does not examine access to care for ADHD |
| Mehta S et al (2009) Unmet prescription medication need in US children. Journal of the American Pharmacology Association 49 (6): 769-776 | No extractable data related to ADHD or its symptoms |
| Merikangas KR et al (2010) Prevalence and treatment of mental disorders among US children in the 2001-2004 NHANES. Pediatrics 125: 75-81 | Less than 50% of the sample has ADHD characteristics |
| Merikangas KR et al (2011) Service utilisation for lifetime mental disorders in US adolescents: Results of the National Comorbidity Survey: Adolescent Supplement. Journal of the American Academy of Child and Adolescent Psychiatry 50 (1): 32-45 | Less than 50% of the sample has ADHD characteristics |
| Miller AR et al (2005) Family physician’s involvement and self-reported comfort and skill in care of children with behavioural and emotional problems: A population based survey. BMC Family Practice 6 (1): 12 | Does not examine access to care for ADHD |
| Mitchell J and Read J (2012) Attention-deficit hyperactivity disorder, drug companies and the internet, Clinical Child Psychology and Psychiatry 17: 121 | Does not examine access to care for ADHD |
| Moen OL et al (2011) Contending and adapting every day: Norwegian parents lived experience of having a child with ADHD. Journal of Family Nursing 17 (4): 441-62 | Sample comprises children with an existing diagnosis of ADHD |
| Monastra VJ (2005) Overcoming the barriers to effective treatment for ADHD: A neuro-educational approach. International Journal of Psychophysiology 58 (1): 71-80 | Sample comprises children with an existing diagnosis of ADHD |
| Moosa F & Lohawala A (2007) ADHD: Do guidelines translate into service? Child and Adolescent Mental Health 12 (2): 73-75 | Does not examine access to care for ADHD |
| Norvilitis JM & Fang P (2005) Perceptions of ADHD in China and the United States: A preliminary study. Journal of Attention Disorders 9 (2): 413-424 | Does not examine access to care for ADHD |
| Odom SE (1996) Effects of an educational intervention on mothers of male children with ADHD. Journal of Community Health Nursing 13 (4): 207-220 | Sample comprises children with an existing diagnosis of ADHD |
| O'Keeffe, N. and McNicholas F (2011). "Paediatricians' views on their role in the assessment and management of ADHD and autism." Irish Medical Journal 104(9) | Does not examine access to care for ADHD |
| Olaniyan O et al (2007) Community Perspectives of Childhood Behavioural Problems and ADHD Among African American Parents. Ambulatory Pediatrics. 7 (3): 226-231 | Does not examine access to care for ADHD |
| Olfson M et al (2003) National trends in the treatment of attention deficit hyperactivity disorder. American Journal of Psychiatry 160 (6): 1071-1077 | Sample comprises children with an existing diagnosis of ADHD |
| Olson BG et al (2005) Improving Guideline adherence for the diagnosis of ADHD in an ambulatory pediatric setting. Ambulatory Pediatrics 5 (3): 138-142 | Does not examine access to care for ADHD |
| Ossebaard HC et al. (2010) A study of a Dutch online decision aid for parents of children with ADHD. Journal of Telemedicine and Telecare 16 (1): 15-19 | Does not examine access to care for ADHD |
| Owens JS et al (2008) Science to practice in underserved communities: The effectiveness of school mental health programming. Journal of Clinical and Child Adolescent Psychiatry 37 (2): 434-447 | No extractable data related to ADHD or its symptoms |
| Palmer NB et al (2010) ADHD and telemental health. Current Psychiatric Reports 12 (5): 409-417 | Not an empirical study |
| Pastor PN & Reuben CA (2005) Racial and ethnic differences in ADHD and LD in young school-age children: Parental reports in the National Health Interview Survey. Public Health Report 120 (4): 383-392 | Does not examine access to care for ADHD |
| Pavuluri, MN et al (1996) [Help-seeking for behavior problems by parents of preschool children: A community study.](http://ovidsp.uk.ovid.com/sp-3.4.2a/ovidweb.cgi?&S=EJFOPDENLCHFHFCDFNBLOHAGNIEHAA00&Complete+Reference=S.sh.16%7c12%7c1) Journal of the American Academy of Child & Adolescent Psychiatry. 35 (2): 215-222 | No extractable data related to ADHD or its symptoms |
| Perrin JM et al (2005) Benefits for employees with children with ADHD: Findings from the Collaborative Employee Benefit Study. Journal of Developmental and Behavioural Paediatrics 26 (1): 3-8 | Does not examine access to care for ADHD |
| Perry CE et al (2005) Latino parents’ accounts of attention deficit hyperactivity disorder. Journal of Transcultural Nursing 16 (4): 312-321 | Sample comprises children with an existing diagnosis of ADHD |
| Pescosolido BA et al (2008) Public knowledge and assessment of child mental health problems: Findings from the National Stigma Study – Children. Journal of the American Academy of Child and Adolescent Psychiatry 47 (3): 339-349 | Does not examine access to care for ADHD |
| Pham AV et al (2010) Ethnic differences in parental beliefs of ADHD and treatment. Journal of Attention Disorders 13 (6): 584-591 | Sample comprises children with an existing diagnosis of ADHD |
| Polaha J et al. (2005) The assessment of ADHD in rural primary care: The portability of the American Academy of Pediatrics Guidelines to the real world. Pediatrics 115 (2): e120-126 | Does not examine access to care for ADHD |
| Porter SC et al (2011) Health literacy and task environment influence parents burden for data entry on child specific health information: randomised control trial. Journal of Medical Internet Research 13 (1): e13 | Does not examine access to care for ADHD |
| Power TJ et al (2008) Managing ADHD in Primary Care: A systematic analysis of roles and challenges. Pediatrics 121 (1): e65-72 | Does not examine access to care for ADHD |
| Rabbani A & Alexander G (2009) Impact of family structure on stimulant use among children with ADHD. Health Services Research 44 (6): 2060-2078 | Sample comprises children with an existing diagnosis of ADHD |
| Rabiner DL et al (2010) Instability in teacher ratings of children’s inattentive symptoms: Implications for the assessment of ADHD. Journal of Developmental and Behavioural Pediatrics 3: 175-180 | Sample comprises children with an existing diagnosis of ADHD |
| Ralfalovich A (2005) Exploring clinician uncertainty in diagnosis and treatment of ADHD. Sociology of Health and Illness 27 (3): 305-323 | Does not examine access to care for ADHD |
| Ray GT et al (2006) Attention-deficit/hyperactivity disorder in children: Excess costs before and after initial diagnosis and treatment cost differences by ethnicity. Archives of Pediatric Adolescent 160 (10): 1063-1069 | Sample comprises children with an existing diagnosis of ADHD |
| Reid R et al (1996) Educating every teacher, every year: the public schools and parents of children with ADHD. Seminars in Speech and Language 17 (1): 73-90 | Sample comprises children with an existing diagnosis of ADHD |
| Reigstad B et al (2004) Changes in referrals to child and adolescent psychiatric services in Norway 1992-2001. Social Psychiatry and Psychiatric Epidemiology 39 (10): 818-827 | No extractable data related to ADHD or its symptoms |
| Ronhovde LI & Ekevik E (2005) Interdisciplinary cooperation important in ADHD. Tidsskr Nor Laegeforen 125 (4): 466 | Not written in English |
| Rossbach M and Probst P (2005) [Development and evaluation of an ADHD teacher group training: A pilot study. Praxis der Kinderpsychologie und Kinderpsychiatrie xx (8): 645-663 | Not written in English |
| Rostain AL et al (1993) Assessing parents’ willingness to pursue treatment for children with attention-deficit hyperactivity disorder Journal of the American Academy of Child and Adolescent Psychiatry 31 (1): 175-181 | Sample comprises children with an existing diagnosis of ADHD |
| Rushton JL et al (2004) Use of practice guidelines in the primary care of children with ADHD. Pediatrics 114 (1): e23-28 | Does not examine access to care for ADHD |
| Salmond CH and Jim J (2007) A characterisation of tier 2 services in child and adolescent mental health. Child and Adolescent Mental Health 12 (2): 87-93 | No extractable data related to ADHD or its symptoms |
| Sarraf et al (2011) A comparative study of the effectiveness of nonattendance and workshop education of primary school teachers on their knowledge, attitude and function towards ADHD students in Isfahan in 2010. Journal of Research in Medical Sciences 16 (9): 1196-1995 | Does not examine access to care for ADHD |
| Schachter et al (2011) Informed consent and stimulant medication: Adolescents and parents ability to understand information about benefits and risks of stimulant medication for the treatment of ADHD. Journal of Child and Adolescent Psychopharmacology 21 (2): 139-148 | Sample comprises children with an existing diagnosis of ADHD |
| Schweifer C (2009) ADHD in school children: Knowledge, resources and cooperation among professionals concerned. Wiener Medizinische Wochenschrift 159 (7-8): 183-187 | Not written in English |
| Shaw K et al (2003) A qualitative study of Australian GPs’ attitudes and practices in the diagnosis and management of Attention Deficit/Hyperactivity Disorder. Family Practice 20 (2) 129-134 | Does not examine access to care for ADHD |
| Shaw KA et al (2002) Attitudes and practices of general practitioners in the diagnosis and management of Attention Deficit/Hyperactivity Disorder. Journal of Pediatric and Child Health 38 481-486 | Does not examine access to care for ADHD |
| Sindelar PT & Meisel CJ (1982) Teacher-physician interaction in the treatment of children with behavioural disorders. International Journal of Partial Hospitalisation 1 (3): 271-277 | Does not examine access to care for ADHD |
| Sitholey, P., V. Agarwal, et al. (2011). "A preliminary study of factors affecting adherence to medication in clinic children with attention-deficit/hyperactivity disorder." Indian Journal of Psychiatry 53(1): 41-44 | Sample comprises children with an existing diagnosis of ADHD |
| Skilling et al (2008) A survey of Attention-Deficit/ Hyperactivity Disorder follow-up. Scottish Medical Journal 53 (2): 12-14 | Does not examine access to care for ADHD |
| Sleath BLE et al (2006) Literacy and perceived barriers to medication taking among homeless mothers and their children. American Journal of Health Systems Pharmacy. 63 (4): 346-351 | Less than 50% of the sample has ADHD characteristics |
| Sleator EK et al (1982) How do hyperactive children feel about taking stimulants and will they tell the Doctor? Clinical Pediatrics 21 (8): 474-479 | Sample comprises children with an existing diagnosis of ADHD |
| Stein REK et al (2008) Do paediatricians think they are responsible for identification and management of child mental health problems? Results of the AAP periodic survey. Ambulatory Pediatrics 8 (1): 11-17 | Less than 50% of the sample has ADHD characteristics |
| Stein REK et al (2009) ADHD: How much responsibility are paediatricians taking? Pediatrics 123 (1): 248-255 | Does not examine access to care for ADHD |
| Stevens J et al (2004) Ethnic and regional differences in primary care visits for attention-deficit hyperactivity disorder. Journal of Developmental and Behavioural Pediatrics 25 (5): 318-325 | Sample comprises children with an existing diagnosis of ADHD |
| Stevens J et al (2005) Race/ethnicity and insurance status as factors associated with ADHD treatment patterns. Journal of child and adolescent psychopharmacology 15 (1): 88-96 | Sample comprises children with an existing diagnosis of ADHD |
| Tamm L et al (2005) Intervention for pre-schoolers at risk for ADHD: service before diagnosis. Clinical Neuroscience Research 5 (5-6): 247-253 | Does not examine access to care for ADHD |
| Tettenborn et al (2008) The provision and nature of ADHD services for children and adolescents in the UK: Results from a nationwide survey. Clinical Child Psychology and Psychiatry 13 (2): 287-304 | Does not examine access to care for ADHD |
| Toomey et al (2011) The patient centred medical home, practice patterns and functional outcomes for children with ADHD. Academic Pediatrics 11 (6): 500-507 | Does not examine access to care for ADHD |
| Toomey SL et al (2008) Does connection to primary care matter for children with ADHD. Pediatrics 122 (2): 368-374 | Sample comprises children with an existing diagnosis of ADHD |
| Vlam SL (2006) ADHD: Diagnostic assessment methods used by APRN. Pediatric Nursing 32 (1): 18-24 | Does not examine access to care for ADHD |
| Wade T et al (2008) Utilization patterns of SBHC at urban and rural elementary and middle schools. Public Health Reports 123 (6): 739-750 | Less than 50% of the sample has ADHD characteristics |
| Walker E et al (1984) Teachers assumptions regarding the severity causes and outcomes of behavioural problems in preschoolers. Implications for referral. Journal of Consulting and Clinical Psychology 52 (5): 899-902 | No extractable data related to ADHD or its symptoms |
| Walter HJ et al (2006) Teachers’ beliefs about mental health needs in inner city elementary schools. Journal of American Academy of Child and Adolescent Psychiatry 45 (1): 61-68 | Does not examine access to care for ADHD |
| Ward R et al (1999) Physician outcomes and implications for planning an intensive educational experience on ADHD. Academic Medicine 74 (10Suppl): S31-3 | Does not examine access to care for ADHD |
| Waschbusch DA et al (2011) A discrete choice conjoint experiment to evaluate parent preferences for treatment of young, medication naive children with ADHD. Journal of Clinical Child and Adolescent Psychology 40 (4): 546-561 | Sample comprises children with an existing diagnosis of ADHD |
| Wheeler L et al (2008) ADHD in schools: Prevalence, multi-professional involvement and school training needs. Emotional and Behavioural Difficulties 13 (3): 163-177 | Sample comprises children with an existing diagnosis of ADHD |
| Wilcox CE et al (2007) Seeking help for ADHD in developing countries: A study of parental explanatory models in Goa, India. Social Science and Medicine 64 (8): 1600-1610 | Sample comprises children with an existing diagnosis of ADHD |
| Wolraich ML et al (2010) Pediatricians attitudes and practices on ADHD before and after the development of pediatric practice guidelines. Journal of Attention Disorders 13 (6): 563-572 | Does not examine access to care for ADHD |
| Zimmerman FJ (2005) Social and Economic Determinants of Disparities in Professional Help-Seeking for Child Mental Health Problems: Evidence from a National Sample. Health Services Research 40 (5 pt 1): 1514-1533 | No extractable data related to ADHD or its symptoms |
